# Integrated Bayesian Approaches Shed Light on the Dissemination Routes of the Eurasian Grapevine Germplasm

**DOI:** 10.3389/fpls.2021.692661

**Published:** 2021-08-05

**Authors:** Francesco Mercati, Gabriella De Lorenzis, Antonio Mauceri, Marcello Zerbo, Lucio Brancadoro, Claudio D'Onofrio, Caterina Morcia, Maria Gabriella Barbagallo, Cristina Bignami, Massimo Gardiman, Laura de Palma, Paola Ruffa, Vittorino Novello, Manna Crespan, Francesco Sunseri

**Affiliations:** ^1^Istituto Bioscienze e Biorisorse, Consiglio Nazionale delle Ricerche, Palermo, Italy; ^2^Dipartimento di Scienze Agrarie ed Ambientali, Università degli Studi di Milan, Milan, Italy; ^3^Dipartimento Agraria, Università Mediterranea degli Studi di Reggio Calabria, Reggio Calabria, Italy; ^4^Dipartimento di Scienze Agrarie, Alimentari e Agro-ambientali, Università degli Studi di Pisa, Pisa, Italy; ^5^CREA - Centro di Ricerca per la Genomica e la Bioinformatica, Fiorenzuola d'Arda, Italy; ^6^Dipartimento di Scienze Agrarie e Forestali, Università degli Studi di Palermo, Palermo, Italy; ^7^Dipartimento di Scienze della Vita, Centro Biogest-Siteia, Università degli Studi di Modena e Reggio Emilia, Reggio Emilia, Italy; ^8^CREA - Centro di Ricerca per la Viticoltura ed Enologia, Conegliano, Italy; ^9^Dipartimento di Scienze Agrarie, Alimenti, Risorse Naturali e Ingegneria, Università degli Studi di Foggia, Foggia, Italy; ^10^Istituto per la Protezione Sostenibile delle Piante, Consiglio Nazionale delle Ricerche, Torino, Italy; ^11^Dipartimento di Scienze Agrarie, Forestali e Alimentari, Università degli Studi di Torino, Grugliasco, Italy

**Keywords:** *Vitis vinifera* L. subsp. *sativa*, SNP array, genetic structure, LD decay, ancestry coefficients, migration events

## Abstract

The domestication and spreading of grapevine as well as the gene flow history had been described in many studies. We used a high-quality 7k SNP dataset of 1,038 Eurasian grape varieties with unique profiles to assess the population genetic diversity, structure, and relatedness, and to infer the most likely migration events. Comparisons of putative scenarios of gene flow throughout Europe from Caucasus helped to fit the more reliable migration routes around the Mediterranean Basin. Approximate Bayesian computation (ABC) approach made possible to provide a response to several questions so far remaining unsolved. Firstly, the assessment of genetic diversity and population structure within a well-covered dataset of ancient Italian varieties suggested the different histories between the Northern and Southern Italian grapevines. Moreover, Italian genotypes were shown to be distinguishable from all the other Eurasian populations for the first time. The entire Eurasian panel confirmed the east-to-west gene flow, highlighting the Greek role as a “bridge” between the Western and Eastern Eurasia. Portuguese germplasm showed a greater proximity to French varieties than the Spanish ones, thus being the main route for gene flow from Iberian Peninsula to Central Europe. Our findings reconciled genetic and archaeological data for one of the most cultivated and fascinating crops in the world.

## Introduction

Grapevine (*Vitis vinifera* L. subsp. *sativa)* is one of the earliest domesticated and most cultivated crops worldwide, having a great impact on the agri-food economy and prized for its fruits and wines. Europe is the largest producer (primarily Italy, Spain, and France), followed by Asia and America. Nowadays, worldwide grape production reaches nearly 78 M tons (http://faostat.fao.org/).

Historical and archaeologica evidence dated grapevine domestication back to the Neolithic age (ca. 8,500–4,000 BC), when human populations began to collect and propagate *Vitis* forms to improve fruit and wine production (McGovern et al., 1986; Zohary and Hopf, 2000). Domestication took place in the region between the Caucasus and Mesopotamia (Georgia, Iran, Turkey), domesticating wild populations of *Vitis vinifera* subsp. *sylvestris*, considered to be the ancestor of the cultivated form, the subsp. *sativa* (Levadoux, 1956; Myles et al., 2011; De Andrés et al., 2012; McGovern et al., 2017). From the oldest domestication sites, cultivated grapevines disseminated westward into neighboring regions (Egypt and Lower Mesopotamia), reaching the Mediterranean Basin, together with the development of human culture. Romans later spread grapevine cultivation in the temperate areas of Europe, following the main trade routes along the major rivers Rhine, Rhone, and Danube. Islam then played an important role in spreading table grapes to Northern Africa, Spain, and Middle East (This et al., 2006).

The Italian Peninsula seems to have had a key role in the spreading of grapevine and viticulture practices from Greece to Central and Western Europe, due to its strategic position on the Mediterranean Sea. In the eighth century BC, the establishment of Greek colonies in the Southern Italy was the main driver of viticulture expansion and development in these areas. In the beginning, the settlers conceivably introduced vines from their places of origin, nonetheless they plausibly also cultivated local populations crossed with Greek varieties (Buono and Vallariello, 2002; McGovern, 2003; Scienza, 2004; Marvelli et al., 2013).

In Europe, a long period of significant successes for viticulture has been witnessed due to the human selection of new and valuable cultivars, up to the current age. To date, the Vitis International Variety Catalog (http://www.vivc.de) and the Italian Register (http://catalogoviti.politicheagricole.it) list more than 13,000 and nearly 800 Eurasian grapevine varieties, respectively.

Genetic diversity and population structure of the cultivated varieties has been extensively assessed throughout highly polymorphic microsatellite markers (simple sequence repeat, SSR) (Cipriani et al., 2010; Laucou et al., 2011; Lacombe et al., 2012; Bacilieri et al., 2013; Emanuelli et al., 2013). Recently, by advanced next-generation sequencing, three high-throughput *Vitis* genotyping chip arrays, including 9, 18, and 37k single nucleotide polymorphism (SNP) loci were developed (Myles et al., 2010; Marrano et al., 2017; Laucou et al., 2018). These new flexible and performing tools became as valuable as SSR in providing grapevine genetic diversity, structure information, pedigree analysis, gene flow, and genome-wide association analysis (GWAS) with higher-throughput and cost-effectiveness than SSRs (Myles et al., 2011; De Lorenzis et al., 2015, 2019; Mercati et al., 2016; Marrano et al., 2017; Laucou et al., 2018; Sunseri et al., 2018).

Despite the large efforts to analyze the genetic variability is available worldwide, a myriad of questions remains unsolved. First, the relationships within and among populations: What is the spreading history of Italian and the other Eurasian populations? What was the role of Caucasian germplasm in shaping the grapevine populations cultivated worldwide? How much did the ancient population affect the shape of the present ones? Does the grapevine genetic variability fit the human migration routes around the Mediterranean Basin?

To address the above questions, a comprehensive study on 384 varieties belonging to the Italian grapevine germplasm and 654 additional varieties belonging to Eurasia were used to unravel the complexity of grapevine germplasm relationships.

The entire panel allowed us to improve the knowledge on genetic diversity among grapevine populations and their dissemination routes by assessing the genetic structure and population relatedness, computing linkage disequilibrium (LD) decay, and ancestry coefficients, as well as, inferring migration events through the evaluation of putative scenarios.

## Materials and Methods

### Sample Collection and Genotype Calling

Three-hundred-eighty and four grapevines (CREA—https://www.crea.gov.it—and AGER consortium—https://www.progettoager.it) showing unique SNP profiles and covering most of the Italian germplasm were selected from 615 genotypes firstly investigated for parentage analysis (D'Onofrio et al., 2021). To provide an overview of grapevine dissemination throughout Eurasia, 654 varieties from the available datasets (De Lorenzis et al., 2015, 2019; Laucou et al., 2018) were included (Supplementary Table 1). All the genotypes were characterized using the 18k SNP array (Laucou et al., 2018). A stringent filtering [SNP NA rate > 1% and minor allele frequency (MAF) <5%; NA rate for each individual >5%; see D'Onofrio et al., 2021] was applied to ultimately obtain an SNP panel (Supplementary Table 1), then the duplicated profiles detected by calculating the pairwise percentage of mismatches between (20% was used as cut-off) individuals were deleted. After filtering, 6,770 high-quality SNPs and 1,038 unique genotypes were retained (Table 1 and Supplementary Table 1). Based on the previous information (Bacilieri et al., 2013; Laucou et al., 2018; De Lorenzis et al., 2019), 1,038 genotypes were distinguished in six groups for geographic origin: (i) Balkans (BALK); (ii) Eastern Mediterranean and Caucasus, Middle and Far East, Russia and Ukraine (EMCA-MFEAS-RUUK); (iii) Iberian Peninsula (IBER); (iv) ITAP-Northern and Center Italy (north-center); (v) ITAP-Southern Italy (south); (vi) Western and Central Europe (WCEUR).

**Table 1:** Grapevine varieties collected across Europe, Caucasus, Middle, and Far East, genotyped by 18k SNP genotyping array and arranged based on their passport data (for a detailed list, see Supplementary Table 1).

**Group code**	**Origin**	**Subgroup name**	**Countries**	**Samples' number**
BALK	Balkans	Eastern Europe	ALB, BGR, HUN, RO	125
		Balkan Peninsula	BIH, CYP, GRC, HRV, MNE, SCG, SVN	
EMCA-MFEAS-RUUK	Eastern Mediterranean and Caucasus	Caucasus and Turkey	AFG, ARM, AZE, GEO, TJK, TKM, TUR, UZB	167
		Near East	ISR, LBN, SYR	
	Middle and Far East	Middle East	IRN	
	Russia and Ukraine	Russia and Ukraine	MDA, RUS, UKR	
IBER	Iberian Peninsula	Iberian Peninsula	ESP, PRT	196
ITAP-north-center	Italian Peninsula	Italian Peninsula—North	ITA	143
		Italian Peninsula—Center	ITA	105
ITAP-south		Italian Peninsula—South	ITA	136
WCEUR	Western and Central Europe	Western Europe	FRA	166
		Central Europe	AUT, DEU, CHE, TCH	
*Total*				*1,038*

### Genetic Diversity and Population Structure

#### Cluster and Principal Coordinates Analyses

Phylogenetic analysis and principal coordinates analysis (PCoA) on both the Italian (384 genotypes) and whole (1,038) grapevine dataset were performed to estimate the overall relationship among the varieties. The distance-based dendrogram with Nei's genetic distance (Nei, 1972), UPGMA algorithm (bootstraps based on 1,000 re-samplings), and PCoA were developed using R/poppr (Kamvar et al., 2015) and R/adegenet packages (Jombart, 2008), respectively.

#### Structure and Discriminant Analysis of Principal Components

The number of genetic pools (*K*) was computed for both datasets using an admixture model performed through fastSTRUCTURE (Raj et al., 2014), using the input files (.bed,.bim, and.fam) generated by PLINKv1.07 (Purcell et al., 2007) and the *structure.py* python script. The best number of model complexity (*K*) was chosen applying the algorithm for multiple choices, *chooseK.py*. To extract the optimum *K*, the prediction error for each *K* was computed by the cross validation (CV) function. Bayesian analyses for both datasets were performed also by discriminant analysis of principal components **(**DAPC) implemented in the R/adegenet. The Bayesian information criterion (BIC) method (Neath and Cavanaugh, 2012) was used to infer the *K-means* clustering. The analyses were performed independent of geographic origin.

#### Wright's Fixation Index and Neighbor-Joining Tree

The Wright's fixation index (*Fst*), a population pairwise index (Wright, 1965), was computed to evaluate the genetic distances among the six groups outlined in Table 1 using R/HierFstat (Goudet, 2005). A neighbor-joining (NJ)-tree based on *Fst*-values was developed with R/adegenet.

### Identity by Descent Analysis and Linkage Disequilibrium Decay

To identify the common ancestry of putative recent, identity by descent (IBD) segments were detected in the whole dataset. Relatedness analysis using IBD estimation was conducted through R/SNPrelate (Zheng et al., 2012) using the Method of Moments (MoM) (Purcell et al., 2007), and an multi-dimensional scaling (MDS) analysis was performed on the *n* × *n* matrix of genome-wide IBD pairwise distances. The shared haplotypes between individuals with unknown relationships were also evaluated, using PLINK with the default setting. The average pairwise IBD between chromosomes from different individuals in the groups was summarized by comparison in a heatmap.

The estimation of LD as the Pearson's squared correlation coefficient (*r*^2^) was calculated between each pair of molecular markers (Zhao et al., 2005) for the six groups (Table 1). To avoid distortions, a representative and equivalent number of samples for each group was defined by the R/corehunter (Thachuk et al., 2009), using BALK population size (125 varieties) as the cutoff. The pairwise LD, *r*^2^ was calculated using PLINK and the parameters, such as *-maf 0.05, ld-window-r2 set to zero, ld-window 99999*, and *ld-window-kb 10000* (Hill and Robertson, 1968). Bins of 100 kb among all pairwise combinations were determined based on the physical distance of each SNP pair.

### Inference of Migration Events

The maximum likelihood (ML)-tree of data collected and a gene flow model among the geographic groups (Table 1) were developed by TreeMix (Pickrell and Pritchard, 2012). Stratified allele frequencies from PLINK were converted into the TreeMix format using the *plink2treemix.py* script and used as input (https://speciationgenomics.github.io/Treemix/; Pickrell and Pritchard, 2012). Forty independent ML searches following the procedure described by Zecca et al. (2020) were performed. The results were filtered based on their likelihood values using the R/cfTrees (Zecca et al., 2020), duplicates were deleted, and the best-scoring ML tree was used. The gene flow model among the groups was investigated through migration events (*m*). Migration edges were tested 10 times from 1 to 5 with different random seeds each time and using blocks (k) of 20 SNPs, to check for convergence in terms of the likelihood value of each model, and the variance explained in each migration event was added. Standard errors (SE) and bootstrap replicates (bootstrap) were used to assess the confidence in the inferred tree topology and the weight of migration events. To automate the choice of the best migration event, an *ad hoc* statistic based on the second-order rate of change in the likelihood weighted by SD was adopted through R/OptM (Fitak, 2019). However, since the true model was considered when the migration edges (*m*) explained 99.8% of the variance in ancestry between groups, only the model showing this cutoff was believed to be the best one (Pickrell and Pritchard, 2012). In addition, only the runs with all statistically significant incorporated migration edges were considered. The residuals from the fitted models chosen for our data were visualized using the R script *plot_resid*.

### Inference of Spatial Population Structure Through Ancestry Coefficient

To assess the population structure and the putative different grapevine spreading routes, a spatial ancestry estimation was performed using R/TESS3 (Caye et al., 2016). The ancestry coefficient was evaluated on the whole dataset by adding 21 genotypes from the Maghreb, named MAGH(Laucou et al., 2018), reaching 1,059 varieties (Supplementary Table 1). The samples from Northern Africa appeared useful to verify their possible role as a bridge between the first domestication center, EMCA-MFEAS-RUUK, and IBER. The MAGH group was not included in the previous analysis due to its small size compared to the other six groups (Supplementary Table 1). The number of the best *K* was chosen after a cross-entropy criterion evaluation for each *K* (Frichot et al., 2014; Caye et al., 2016). The values of Q-matrix for the best *K* were interpolated on a geographic map.

### Approximate Bayesian Computation Analysis

An approximate Bayesian computation (ABC) approach implemented with DIY-ABC 2.0 software was adopted to establish the most likely grapevine gene flow scenarios (Cornuet et al., 2014). The genotypes belonging to the six groups (Table 1) and Maghreb were used (Supplementary Table 1). Four groups of hypotheses were evaluated.

#### About the Origin of Southern Italian Genotypes

The first hypothesis aimed to clarify the origin of Southern Italy germplasm, assuming that grapevine was spread from Greek shores to the Southern Italy and then to the Central and Northern Italy (Buono and Vallariello, 2002; Scienza, 2004; Myles et al., 2011; Bacilieri et al., 2013; Riaz et al., 2018; De Lorenzis et al., 2019). The three tested scenarios are as follows: (i) Scenario 1 assumed a flow of domesticated genotypes from BALK into ITAP-south, and then into ITAP-north-center; (ii) Scenario 2 assumed that domesticated grapevines from BALK first flowed into ITAP-north-center, then to ITAP-south; (iii) Scenario 3 assumed that ITAP-south and ITAP-north-center germplasm derived independently from BALK.

#### The Gene Flow From Italy to the Western and Central Europe

The second hypothesis took into account the gene flow from Italy to the Western and Central Europe to dissect the relationship among the Northern Italian and the Western-Central European germplasm (De Lorenzis et al., 2019). Three scenarios were depicted: (i) Scenario 4 assumed that ITAP-south germplasm spread in the Northern-Central Italy and then WCEUR; (ii) Scenario 5 assumed that ITAP-south germplasm spread first in WCEUR and then in ITAP-north-center; (iii) Scenario 6 assumed that an admixture event between ITAP-south and WCEUR genotypes flowed into ITAP-north-center germplasm.

#### The Gene Flow From ITAP-South to WCEUR Through IBER

The third case proposed the hypothesis of a gene flow from the Southern Italy to WCEUR *via* IBER (Buono and Vallariello, 2002); The three supposed scenarios are as follows: (i) Scenario 7 assumed that grapevine germplasm from ITAP-south was introduced first into IBER and then into WCEUR; (ii) Scenario 8 assumed that WCEUR genotypes derived from an admixture event between ITAP-south and IBER genotypes; (iii) Scenario 9 assumed the contrary hypothesis of Scenario 7.

#### The Gene Flow From Middle and Far East to IBER Through Northern Africa

Lastly, the hypothesis of a gene flow from the Middle and Far East to IBER through Northern Africa was verified. The three scenarios were as follows: (i) Scenario 10 assumed a gene flow from EMCA-MFEAS-RUUK to Northern Africa (MAGH) and then to IBER (ii) Scenario 11 assumed that an admixture event between MAGH and EMCA-MFEAS-RUUK germplasm flowed into the IBER genotypes; (iii) Scenario 12 assumed a contrary hypothesis to Scenario 10.

#### Calculation Setting Up, Core Collections, and Statistics

To reduce the computational power required to perform the ABC analysis, the dataset were pruned with PLINK, removing SNP loci pairs not in optimal LD, using a sliding window of 50 bp and a step size of 5 SNPs, with a variance inflation factor 2 (Orozco-terWengel et al., 2015). A core collection was also built for each population using the allele coverage allocation strategy implemented in R/corehunter, which are useful to maximize the allele proportion. One hundred thousand datasets were simulated by do it yourself (DIY)-ABC, for a total of 12 × 10^5^ simulated datasets, considering all scenarios. Mean gene diversity across all loci, mean across loci of *Fst* distances between pairs of geographic groups, and mean across loci of *Nei's* genetic distances between pairs of geographic groups were calculated. The most appropriate scenario for each hypothesis was selected comparing the summary statistics of simulated and observed datasets. The posterior probabilities [with 95% confidence interval (CI)] of each competing scenario were also estimated following a logistic regression approach on the 1% of simulated datasets closest to the observed dataset. The ability of ABC approach to discriminate between scenarios (ABC performance) was evaluated. Per each scenario, Type I and II error probabilities were also estimated (Cornuet et al., 2014).

Lastly, to validate the goodness-of-fit of the selected scenario, a model checking test was performed, carrying out the local linear regression on the 1% of closest simulated datasets, after adopting a logit transformation to parameters, as suggested by the DIY-ABC user manual (Cornuet et al., 2014).

## Results

### Italian Germplasm Genetic Diversity

#### Phylogenetic and PCoA Analyses

**Figure 1:**
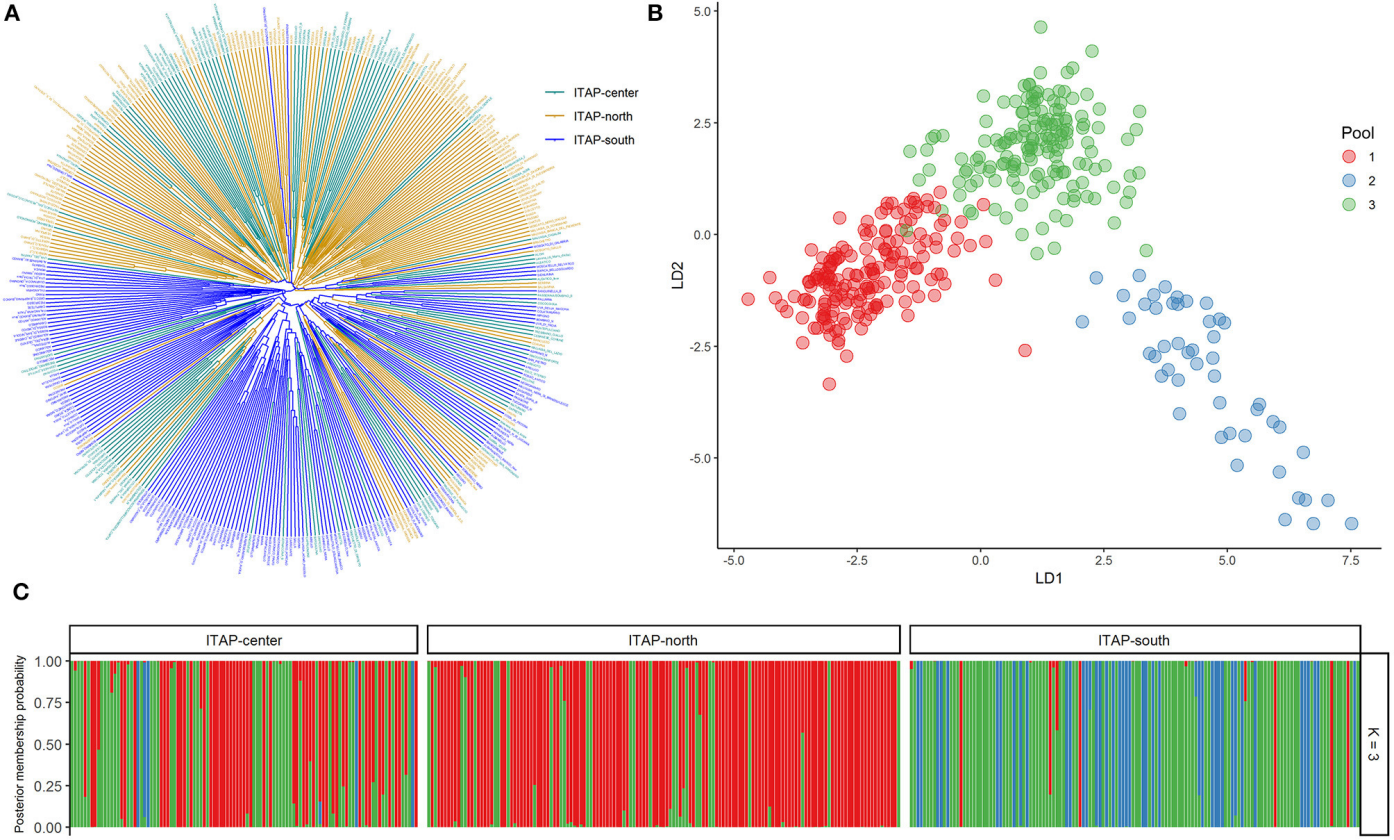
Genetic diversity and population structure on 384 Italian grapevine genotypes analyzed by 18k SNP array. **(A)** UPGMA dendrogram. The varieties were highlighted based on their geographical origin. **(B)** DAPC scatter plot based on the first and second (LD) at the best *K* (3). **(C)** Bar plot of the posterior probability for the best *K* (3) of group assignment for each sample belonging to different origins. UPGMA, unweighted pair group method with arithmetic mean; DAPC, discriminant analysis of principal components.

Phylogenetic analysis among the Italian genotypes highlighted two main groups showing the distinction between Southern and Northern Italian (ITAP-south and -north) varieties, except for 14% of ITAP-south that clustered in the Northern Italian group and *vice versa* for about 16% of the samples (Figure 1A). The Central Italian (ITAP-center) samples were divided between Northern (57%) and Southern (43%) clusters (Figure 1A). A PCoA analysis confirmed the genetic relationships within the Italian germplasm (Supplementary Figure 1), splitting ITAP-north and ITAP-south genotypes into two groups, with the ITAP-center samples divided between them.

#### Structure and DAPC Analysis

The optimum *K* was firstly evaluated with fastSTRUCTURE, providing an optimal value between 2 and 6 (Model complexity that maximizes the marginal likelihood = 6; Model components used to explain structure in data = 2). Based on the prediction error, the lowest model complexity within the range was explained at *K* = 5 (Supplementary Figure 2A).

The DAPC analysis was then performed to further investigate the Italian germplasm and to confirm the group assignments. *K*-means clustering was estimated using the BIC score, defining the best cluster number at *K* = 3 (Supplementary Figure 3 and Supplementary Table 2). The scatter plot highlighted an ITAP-north varieties cluster (mainly gathered in Pool 1, red) and two clusters (Pools 2 and 3, blue and green) including ITAP-center and ITAP-south genotypes, respectively (Figure 1B). The posterior probability at *K* = 3 underlined that nearly all ITAP-south genotypes shared both Pools 2 and 3; the ITAP-center samples split in Pools 1 and 3, while ITAP-north group mainly forms Pool 1 (Figure 1C).

In detail, 196 genotypes (51%) were attributable to a specific pool with a high percentage (ancestry membership >70%) by fastSTRUCTURE. About sixty-two (87%) out of 71 ITAP-north samples formed Pool 1, the ITAP-center genotypes were mainly split between Pools 1 (28) and 3 (19), while Pool 2 is formed almost only by the ITAP-south genotypes (88%), and four from ITAP-center (Supplementary Table 3). About 55% of ITAP-south genotypes were characterized by Pool 3, appearing as a bridge among the Italian varieties (Figure 1B and Supplementary Table 3).

In agreement, DAPC analysis assigned 378 out of 384 (98.4%) Italian varieties to one of the three major pools, setting a cut-off of 70% posterior membership probabilities (Supplementary Table 3). Almost all the Italian varieties were assigned to a pool, with the highest percentage (99%) for both the ITAP-north (141 out of 143) and ITAP-south (135 out of 136) groups, according to Bayesian analysis (Figure 1C and Supplementary Table 3). Although the best model was fixed at *K* = 3 (Figure 1), the same trend in the distribution of Northern, Central, and Southern Italian genotypes was also shown at *K* = 5, based on the cross-validation function (Supplementary Figure 2).

The DAPC results matched with that of PCoA and the unweighted pair group method with arithmetic mean (UPGMA). According to these results, the Northern and Central Italian genotypes were joined and named ITAP-north-center (Supplementary Table 1).

### Genetic Relationship Among Eurasian Grapevine Populations by the Whole Dataset

#### Unweighted Pair Group Method With Arithmetic Mean and PCoA Clustering

**Figure 2:**
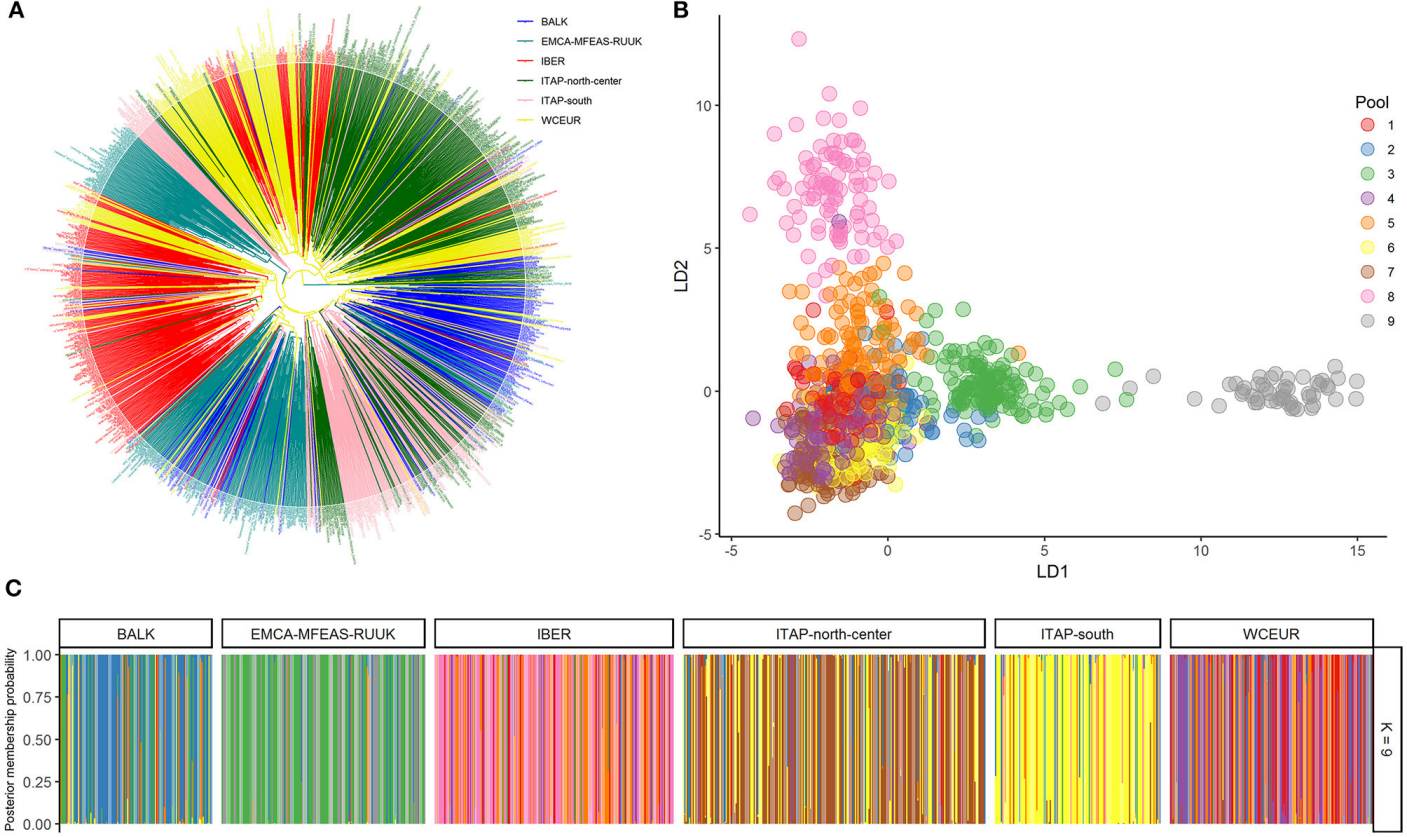
Genetic diversity and population structure on 1,038 grapevine accessions collected across Europe, Caucasus, Middle, and Far East, genotyped by 18k SNP array. **(A)** UPGMA dendrogram; the varieties were highlighted based on their geographical origin (Table 1 and Supplementary Table 1). **(B)** DAPC scatter plot based on the first and second LDs at the best K (9). **(C)** Bar plot of the posterior probability of group assignment for each accession analyzed. The samples are grouped by origin. UPGMA, unweighted pair group method with arithmetic mean; DAPC, discriminant analysis of principal components.

Cluster analysis distinguished genotypes from the IBER, ITAP-north-center, and WCEUR in three clusters (Figure 2A). Nearly 50 Iberian samples (~25%) clustered with WCEUR genotypes. The ITAP-south, BALK, and EMCA-MFEAS-RUUK samples were grouped in a major assorted cluster, where a sub-cluster of ITAP-north-center genotypes was included (Figure 2A, lower part). The ITAP-south varieties appeared more closely related to BALK than EMCA-MFEAS-RUUK genotypes. Another significant cluster included EMCA-MFEAS-RUUK and ITAP-south genotypes, in the upper part of the dendrogram, as a bridge between IBER and WCEUR populations (Figure 2A).

A similar distribution was obtained through PCoA analysis (Supplementary Figure 4), with the whole set divided into three main groups, with some expected overlapping: ITAP-north-center samples grouped together with WCEUR genotypes and separated by Principal Coordinate (PCo) 1; EMCA-MFEAS-RUUK samples clustered together with BALK and ITAP-south groups resulting in complete separation from IBER varieties by PCo2 (Supplementary Figure 4).

#### Structure and DAPC

The optimum pool number was recorded at *K* = 9, using both prediction error and BIC method (Supplementary Table 2 and Supplementary Figure 5). Each population showed the prominent membership (>0.4) to an owner pool computed through both fastStructure and DAPC, except WCEUR (Supplementary Table 4). Moreover, Pool 5 is not owned by any population, with the highest membership related to Moscato bianco (>0.99), the ancestor of muscat flavored varieties (Cipriani et al., 2010; Ruffa et al., 2016). Pool 5 grouped varieties belonging to different populations, showing first- or second-degree relatedness with Moscato bianco (Supplementary Table 1).

The DAPC scatter plot showed a triangle-shaped distribution, highlighting that Pool 9 is divergent from the others (Figure 2B). This pool mainly encompassed genotypes belonging to the EMCA-MFEAS-RUUK population (58%, 98 out of 167 genotypes), while the remaining 30% showed the highest membership with Pool 3. Pools 2 and 3 encompassed the BALK germplasm with 60% (75 out of 125) and 20%, respectively (Figure 2 and Supplementary Table 4). The ITAP-south population showed a unique genetic structure (73%, 100 out of 136) in Pool 6, according to DAPC results (Figure 2C and Supplementary Table 1). All the other populations were split into at least two main pools: ITAP-north-center genotypes shared Pools 6 and 7, IBER population belonged to Pools 5 and 8, while WCEUR genotypes encompassed Pools 1, 4, and 5, the last in common with IBER (Figure 2 and Supplementary Table 4).

### Fixation Index, IBD, and LD Decay

**Figure 3:**
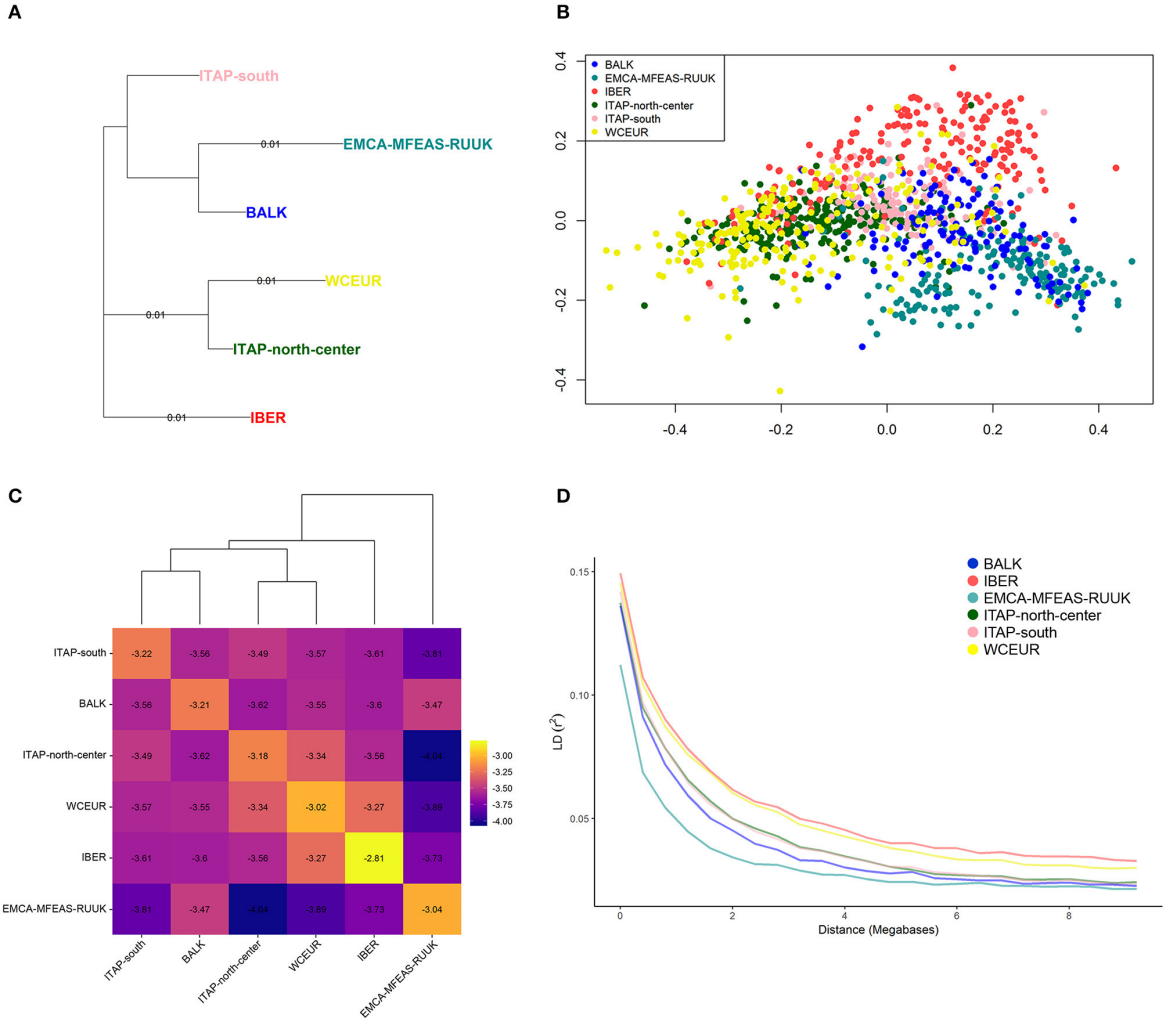
**(A)** NJ-tree based on population pairwise *Fst*. Genetic distances were computed among populations **(**Table 1 and Supplementary Table 1**)**. **(B)** MDS analysis using IBD segments evaluated on 1,038 grapevine varieties, collected across Europe, Caucasus, Middle, and Far East. The varieties were highlighted based on their geographical origin **(**Table 1 and Supplementary Table 1**)**. **(C)** Heatmap of the log average proportion of genome shared IBD between a pair of individuals belonging to groups analyzed. Colors are described in the palette on the right; higher values (less negative, toward the yellow end of the color scale) indicate higher IBD sharing. Cluster obtained through the proportion of shared IBD was also visualized. **(D)** Decay of average LD (*r*^2^) over distance (Mb) in the six groups **(**Table 1**)**. NJ, neighbor-joining; fst, fixation index; MDS, multi-dimensional scaling; IBD, identity by descent; LD, linkage disequilibrium.

The EMCA-MFEAS-RUUK and WCEUR germplasm showed the highest genetic distance according to pairwise *Fst-*values (0.033; Supplementary Table 5). Large genetic divergences were observed between EMCA-MFEAS-RUUK and IBER, as well as ITAP-north-center groups (*Fst* = 0.024). The lowest *Fst* pairwise-value (0.009) was recorded between WCEUR and ITAP-north-center groups, followed by EMCA-MFEAS-RUUK and BALK groups (0.011) and ITAP-south and BALK(0.011) (Supplementary Table 5). The NJ tree developed by *Fst-*values (Figure 3A) split up all the populations showing a structure consistent with previous results. The EMCA-MFEAS-RUUK and BALK groups formed one branch and differed greatly from WCEUR and ITAP-north-center groups, both included in another main branch. The IBER and ITAP-south populations are separated, with the last closely related to BALK samples, in agreement with PCoA (Supplementary Figure 4).

Based on genome-wide IBD pairwise distances, the MDS analysis showed a distinctness of IBER (red), as well as EMCA-MFEAS-RUUK (cyan), and BALK (blue) populations, while the other populations were not clearly separated (Figure 3B). The average amount of IBD shared between individuals in the six populations underlined the relatedness between BALK and ITAP-south genotypes, as well as between ITAP-north-center and WCEUR genotypes, while IBER and EMCA-MFEAS-RUUK populations showed the most consistent genetic distances (Figure 3C).

The average LD declined with the increase of physical distance between markers and appeared fast in all the populations. The fastest decay was observed in EMCA-MFEAS-RUUK population, followed by BALK and in the pair from ITAP (Figure 3D). In contrast, a relatively slower LD decay was recorded for WCEUR and IBER. Thus, the LD decay rate decreased with the increase of geographic distance from the Middle and Far East to the Iberian Peninsula, in an East-to-West gradient. The average distance at which the LD-value reached 0.05, varied among populations, indeed the distance accounted for ~3.2 Mb in the varieties belonging to IBER population, while it decreased to ~1 Mb and 1.8 Mb in the EMCA-MFEAS-RUUK and BALK populations, respectively (Figure 3D).

### Gene Flow Evaluation Through the Estimation of Possible Migration Events

**Figure 4:**
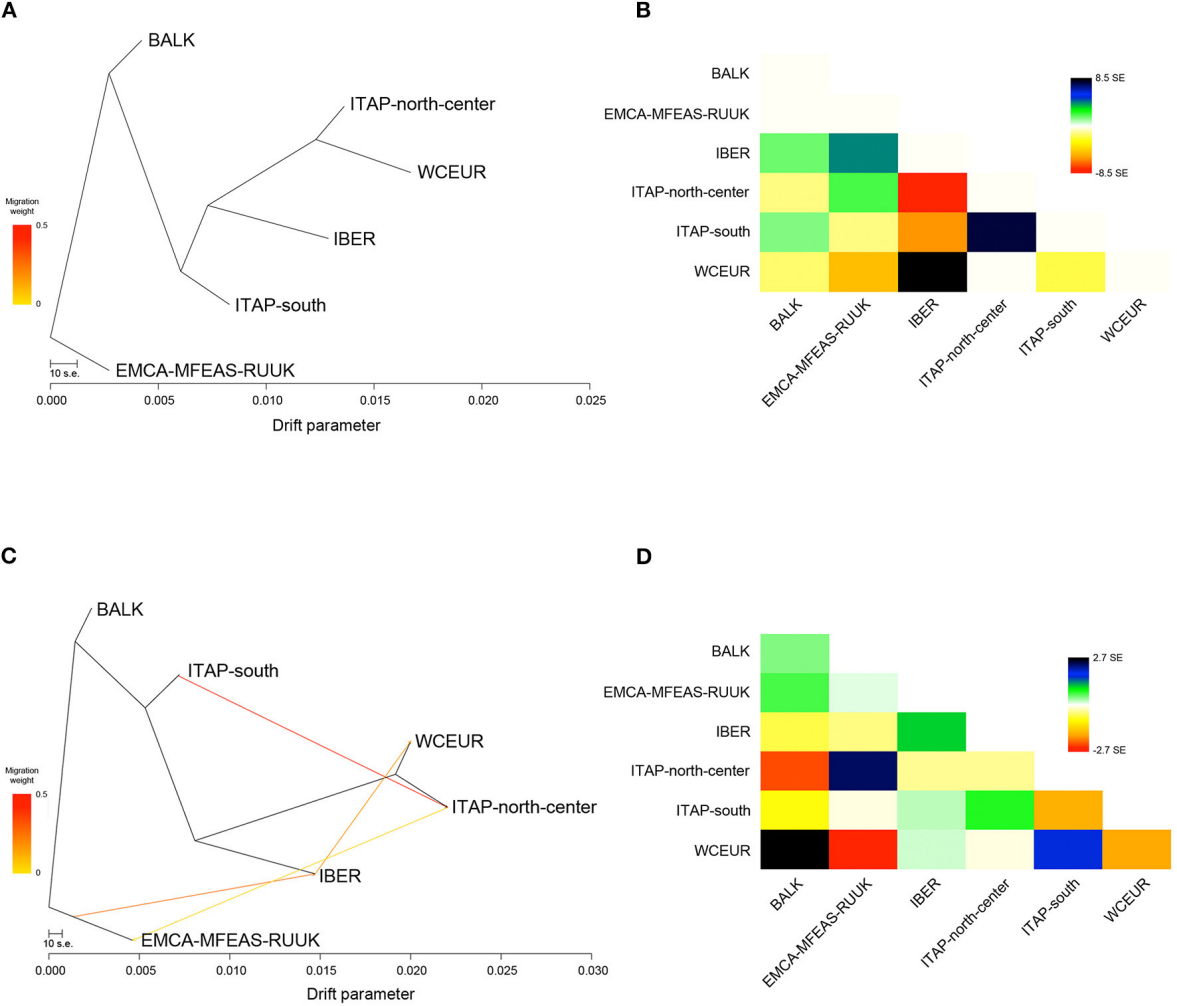
**(A)** The ML-tree inferred by TreeMix. The scale bar shows 10 times the average SE of the entries in the sample covariance matrix. Drift parameter is shown on the x-axis. **(B)** Residual fit from ML-tree with SE ± 8.5. High standard errors suggest strong candidate groups for admixture events. Positive residuals characterize groups where the model underestimates the observed covariance; similarly, pairs of groups where the model overestimates the observed covariance showed negative residuals. Colors are described in the palette on the right. **(C)** ML-tree that best fits the data with 4 additional migration edges: EMCA-MFEAS-RUUK to IBER (*p* < <0.001), ITAP-south to ITAP-north-center (*p* < <0.001), IBER to WCEUR (*p* < <0.001), and ITAP-north-center to EMCA-MFEAS-RUUK (*p* < 0.001). Migration arrow is colored according to its weight and colors are explained in the palette on the left. **(D)** Residual fit from ML-tree at *m* = 4, with SE ± 2.7. ML, maximum likelihood; SE, standard error.

To assess the complex demographic history of the cultivated grapevine, a migration map of ancestral populations based on gene flow was drawn (Figure 4). Forty preliminary ML searches showed the same likelihood (ln)-value, with a similar profile in terms of topology and branch lengths; therefore one ML was chosen randomly. The ML-tree explained 98% of the variance in relatedness between grape populations from different geographic origins, supporting the tree-like history, with branch lengths proportional to the drift amount from the group split (Figure 4A; Supplementary Figure 6A; and Supplementary Table 6). Furthermore, the residuals from the fit of the selected model highlighted a not complete explanation of different population ancestry (Figure 4B). Therefore, five migration events (*m*) were added in sequence, showing the saturation of the model likelihood at four additional migration edges (99.8% of variance explained; Supplementary Figure 6A). Overall, each run at *m* = 4 showed an improvement over the tree without migration, and the model with the highest significance level (*p* < 0.001) for all migration edges recorded was chosen with the best fits at four inferred migration events (Supplementary Table 7). These events were from ITAP-south to ITAP-north-center (weight 44%, *p* < <0.001), from EMCA-MFEAS-RUUK to IBER (weight 27%; *p* < <0.001), from IBER to WCEUR (weight 18%; *p* < <0.001), and from ITAP-north-center to EMCA-MFEAS-RUUK (weight 4%; *p* < 0.001) groups (Figure 4C).

Finally, the optimal number of *m* was also verified through the Evanno and linear methods, inferring two admixture events from ITAP-south to ITAP-north-center (weight 44%; *p* < <0.001) and from EMCA-MFEAS-RUUK to IBER (weight 35%; *p* < <0.001) populations (Supplementary Figures 6B,C, 7A and Supplementary Table 6). Comparing both explained variances and residual fit between the two possible models, four migrations or two admixture events, the best one of *m* = 4 with the highest *f* (0.998 vs. 0.992) and the lowest residual values (SE ± 2.7 vs. SE ± 6.8) (Figure 4D; Supplementary Figure 7B; and Supplementary Table 6) were obtained.

### Grapevine Spreading Evaluation Through Ancestry Coefficient Analysis

The ancestry estimation was performed adding 21 Northern African (MAGH) genotypes (Laucou et al., 2018) to the whole dataset to better overview the conceivable grapevine migration routes.

**Figure 5:**
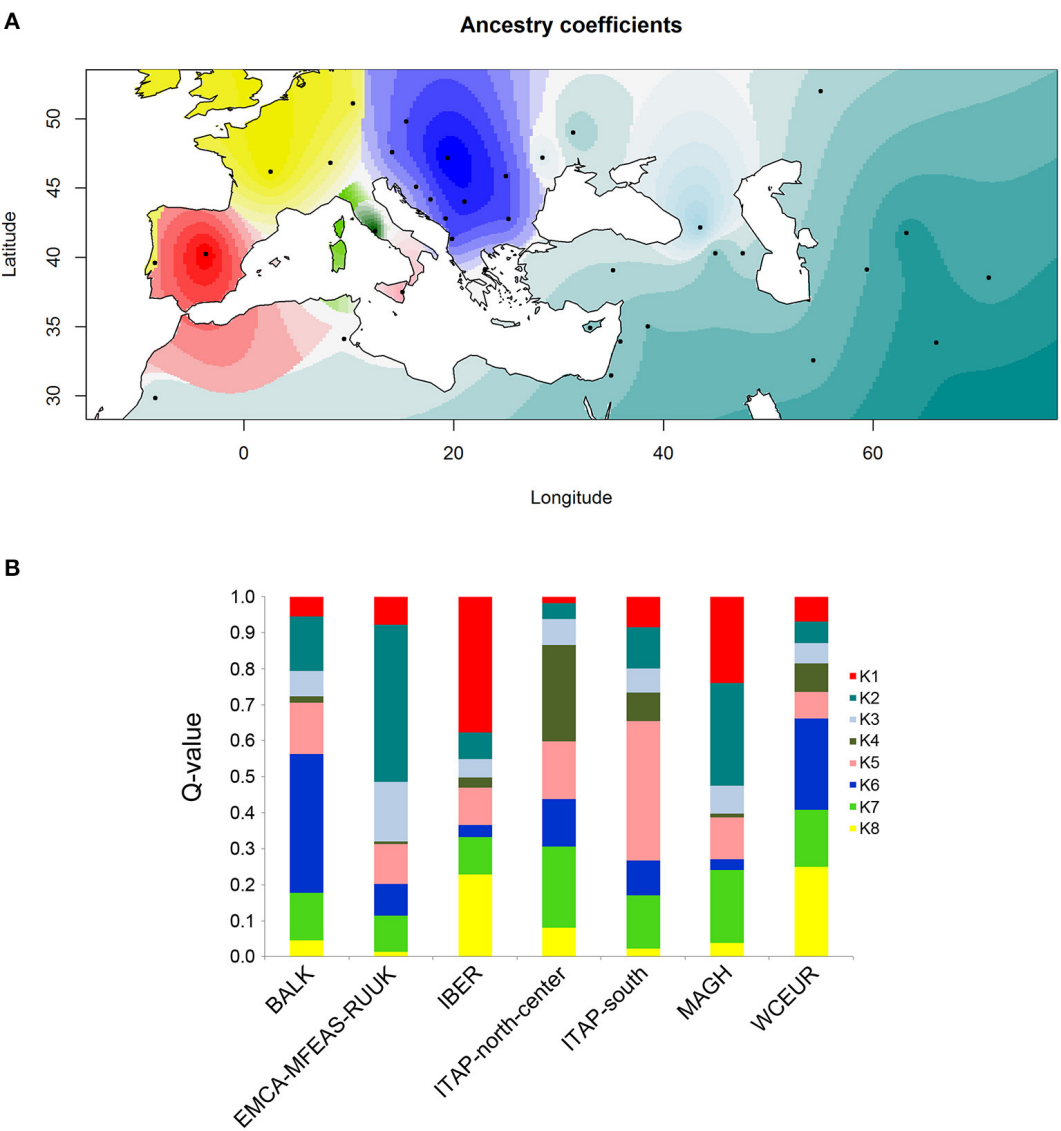
Ancestry coefficient evaluated by R/TESS3 for the whole grapevine germplasm including 21 samples from North Africa (Morocco and Tunisia) named MAGH. The number of ancestral populations (*K* = 8) was chosen after the evaluation of a cross-entropy criterion for each *K* **(**Supplementary Figure 9**)**. **(A)** The values of the Q-matrix (*K* = 8) were interpolated on a geographic map, using the centroid for each country studied, except for Russia for a better graphic display. Likewise, the samples from Bosnia and Herzegovina, Croatia, Montenegro, and Serbia were grouped together for a clearer visualization. **(B)** Bar plot showing the ancestry proportions contributed by *K* ancestral source populations by calculating the average value of each of the 8 membership coefficients (*Q*-value) for each of the seven groups defined by geographic origin **(**Table 1 and Supplementary Table 1**)**.

The analysis of ancestry through the cross-entropy criterion on the total 1,059 varieties estimated a single fixed *K* = 8 (Supplementary Figure 8), confirming the distinctness among grapevine populations as revealed above (Figure 5A).

The ancestry coefficients interpolated to the geographic coordinates clearly showed the grapevine spreading from the first domestication center (Caucasus) to the Mediterranean Basin (Figure 5A). Moreover, four out of the seven grapevine populations resulted in the ancestors of a specific pool, except ITAP-north-center, WCEUR and MAGH populations, showing an admixed structure (Figure 5B and Supplementary Table 8).

### The Best Scenarios for East-To-West Grapevine Migration Routes

**Figure 6:**
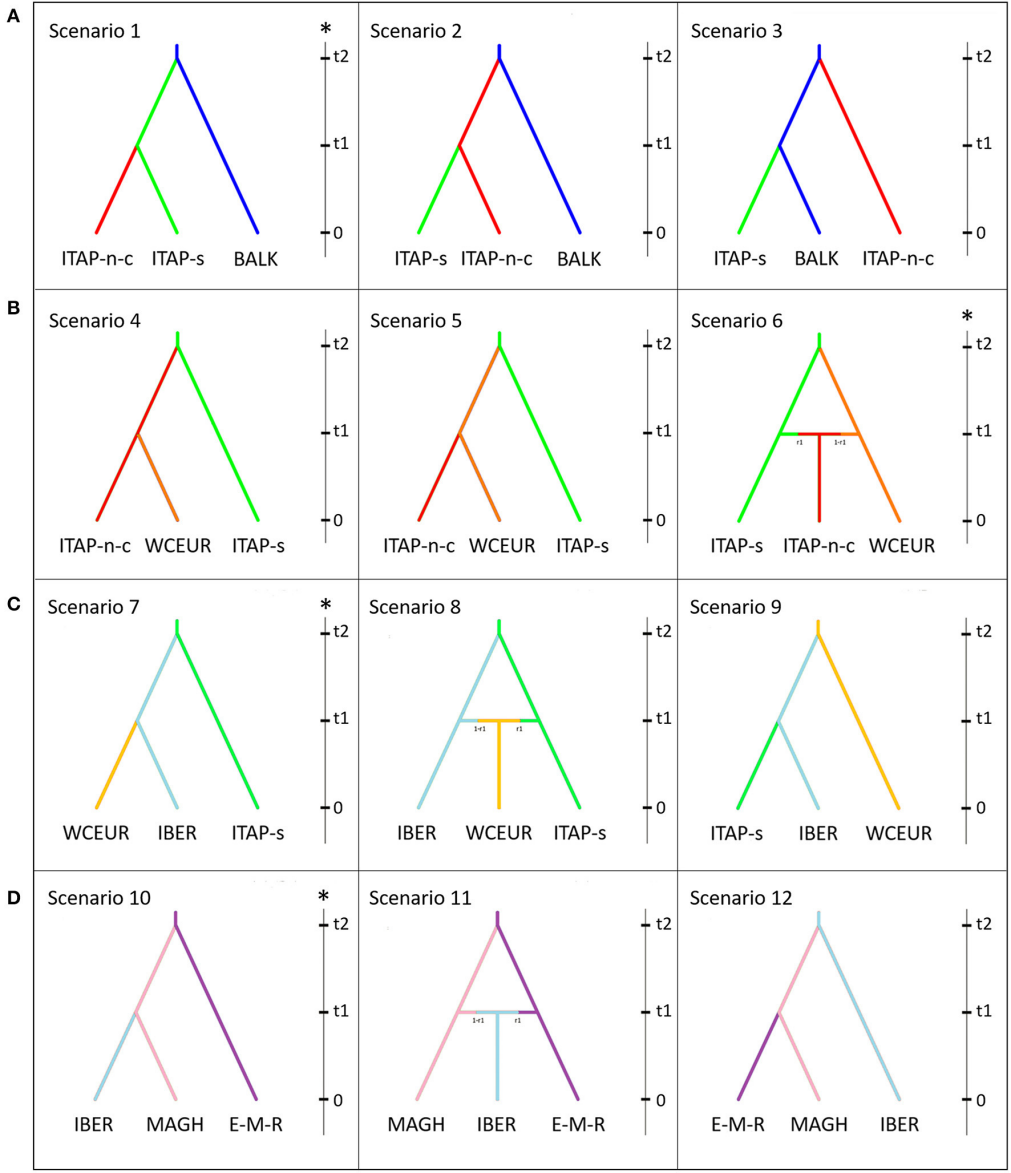
Schematic representation of scenarios depicting the grapevine gene flow in the Mediterranean basin, based on ABC approach. Scenarios were depicted to validate or rebut the gene flow. **(A)** from Balkans to Northern Italy, **(B)** from ITAP-south to Western and Central Europe, **(C)** from ITAP-south to WCEUR through IBER, and **(D)** from Middle and Far East to IBER through Northern Africa. The vertical bar in each box indicates the timescale (*t*_2_ > *t*_1_ > *t*_0_; *t*_0_ = present). For each hypothesis, the most likely scenario (*) was selected among the ones showing: (i) the highest values of logistic regression; (ii) the lowest number of outlying statistics; (iii) the highest value of posterior probability; (iv) the highest value of performance evaluation; (v) the mean values of Types I–II error lower than 20%, indicating an adequate power and sensitivity of ABC analysis (Chen et al., 2017). The samples named EMCA-MFEAS-RUUK **(**Table 1**)** was indicated as E-M-R, while ITAP-north-center and ITAP-south was named, ITAP-n-c and ITAP-s, respectively. ABC, approximate Bayesian computation; EMCA-MFEAS-RUUK; Eastern Mediterranean and Caucasus, Middle and Far East, Russia and Ukraine; ITAP-south, ITAP-Southern Italy; ITAP-north-center, ITAP-Northern, and Center Italy; WCEUR, Western and Central Europe.

Four groups of scenarios were tested using ABC approach to define the possible East-to-West grapevine migration routes (Figure 6 and Supplementary Figure 9). The ABC approach was performed on a pruned SNP dataset (2k) and a core collection per each population, to maintain the available genetic diversity: 21, 31, 30, 33, 34, 37, and 32 genotypes were chosen for MAGH, BALK, EMCA-MFEAS-RUUK, IBER, ITAP-north-center, ITAP-south, and WCEUR groups, respectively.

#### The Origin of ITAP-South Germplasm

Scenario 1 showed the best fitting values, assuming a gene flow from BALK to ITAP-south, then to ITAP-north-center (Figure 6A). For Scenario 1: (i) the highest logistic regression values were recorded (Supplementary Figure 9A); (ii) the posterior probability (Pp)-value was much higher than the others, showing a mean around 74%, with a 95% CI (72–79%) not overlapped with those of the other scenarios; (iii) the lowest number (3 out of 9) of statistics deviated significantly from the observed values (Table 2 and Supplementary Table 9). The ABC performance was of 77% and Type I and Type II error rates were <20% (Supplementary Table 10). Scenario 1 was also confirmed as the most likely one by model check analysis (Supplementary Figure 10A).

**Table 2:** Summary of ABC results to define the most likely scenarios belonging to four hypotheses about the gene flow of grapevine genetic resources from East-to-West.

**Hypothesis 1**	***Scenario 1***	**Scenario 2**	**Scenario 3**
Pp (%)	***74***	51	43
95% CI	***93–95***	48–54	40–45
Number of outlying statistics		
*P* < 0.05	***1***	6	2
*P* < 0.01	***2***	0	3
**Hypothesis 2**	***Scenario 4***	**Scenario 5**	**Scenario 6**
Pp (%)	52	57	***81***
95% CI	46–53	55–64	***75–82***
Number of outlying statistics		
*P* < 0.05	0	2	***1***
*P* < 0.01	4	2	***2***
**Hypothesis 3**	***Scenario 7***	**Scenario 8**	**Scenario 9**
Pp (%)	***88***	57	38
95% CI	***82–91***	54–64	32–40
Number of outlying statistics		
*P* < 0.05	***1***	2	2
*P* < 0.01	***2***	2	2
**Hypothesis 4**	***Scenario 10***	**Scenario 11**	**Scenario 12**
Pp (%)	***75***	46	58
95% CI	***71–79***	42–48	57–60
Number of outlying statistics		
*P* < 0.05	***2***	3	3
*P* < 0.01	***2***	4	2

*Scenarios are defined in Figure 6. Pp, posterior probability; CI, confidence interval. Scenarios with highest Pp-value and lowest number of outlying statistics within each hypothesis are in bold and italic*.

#### Admixture Between ITAP and WCEUR Populations

The second question related to the gene flow between ITAP and WCEUR populations (Figure 6B) established Scenario 6 as the best one, due to the highest logistic regression values, assuming that an admixture event between ITAP-south and WCEUR genotypes flowed into ITAP-north-center germplasm (Supplementary Figure 9B). Scenario 6 showed the highest and significant Pp-value (81%), with a 95% CI ranging from 75 to 82%, 3 out of 9 statistics outlying from the observed values (Table 2 and Supplementary Table 9), 87% of performance evaluation, and Type I and Type II error rates <20% (Supplementary Table 10). The model check analysis confirmed the goodness-of-fit of Scenario 6 (Supplementary Figure 10B).

#### The Gene Flow From ITAP-South to WCEUR Populations Through IBER Population

Among the scenarios hypothesized to evaluate the possibility of a gene flow from ITAP-south to WCEUR population through IBER population (Figure 6C), Scenario 7 showed the highest logistic regression values, assuming that grapevine germplasm from ITAP-south was firstly introduced into IBER and then into WCEUR (Supplementary Figure 9C). Scenario 7 showed the highest Pp rate (88%, with 95% CI from 82 to 91%), the lowest number of outlying statistics (3 out of 9) (Table 2 and Supplementary Table 9), the highest performance evaluation value (92%), and Type I and Type II error rates <20% (Supplementary Table 10). Scenario 7 was confirmed as the most likely by model check analysis (Supplementary Figure 10C).

#### The Gene Flow From EMCA-MFEAS-RUUK to IBER Populations Through MAGH Population

The last ABC test confirmed Scenario 10 as the most likely model (Figure 6D) taking into account the highest rate of logistic regression and assuming a gene flow from EMCA-MFEAS-RUUK to Northern Africa (MAGH) and then to the IBER population (Supplementary Figure 9D). Scenario 10 showed 75% Pp (with 95% CI from 71 to 79%), the lowest number of outlying statistics (4 out of 9), high value of performance evaluation (88%), and Type I and Type II error rates <20% (Table 2, Supplementary Tables 9, 10). The PCA analysis performed in the model check analysis confirmed Scenario 10 as the scenario well-fitting the observed dataset (Supplementary Figure 10D).

## Discussion

Significant efforts were made over the last decades to clarify the complex puzzle of grapevine germplasm evolution and spreading in Eurasia. To trace the history of a perennial crop, the evolution rate should be considered. In grapevine, it is expected to be slow due to the limited sexual reproduction and clonal propagation (Miller and Gross, 2011; Lacombe et al., 2012). Archaeological evidence suggested that grapevine domestication took place in the Near East between the Black and Caspian Sea, which has been confirmed by the genetic analysis (Arroyo-García et al., 2006; This et al., 2006; Myles et al., 2011; McGovern et al., 2017). Moreover, the grapevine domestication appeared dynamic in space and time, defining a continuum from exploited wild to cultivated populations, *via* incipient domesticated populations (Miller and Gross, 2011).

In the grapevine, the coexistence of wild populations and domesticated varieties is well-documented with a bidirectional gene flow (De Andrés et al., 2012; Riaz et al., 2018; D'Onofrio, 2020; Maraš et al., 2020). The consequent generations overlapping due to hybridization events between wild and modern varieties implied stratification that cannot be resolved on a time scale, as revealed by pedigree analysis (Lacombe et al., 2012; D'Onofrio, 2020). Furthermore, the synonymies and homonymies in the cultivated germplasm and the difficulty in assigning the varieties to specific areas made the gene flow studies even more complex. Grapevine is propagated by cuttings; to propagate desirable cultivars indefinitely, these cuttings were introduced into different regions, following human migrations, and were renamed locally (This et al., 2006). In the last few decades, a massive clearing up of synonyms and homonyms and pedigree reconstruction by using molecular markers (SSR, SNP) became necessary. Moreover, the availability of this information on public databases provided advanced knowledge on grapevine varieties supporting their assignment to geographic areas, supplying a decisive input to gene flow studies.

A great deal of information from a large international cultivars panel was recently obtained, although fragmented due to the use of different genotype panels as well as by molecular marker sets (This et al., 2004; Cipriani et al., 2010; Myles et al., 2011; Cunha et al., 2016; Laucou et al., 2018; Riaz et al., 2018; De Lorenzis et al., 2019; D'Onofrio et al., 2021). The relationships within the grapevine germplasm were drawn worldwide, even though not completely. Here, we merged the grapevine genetic resources from two Italian projects (AGER and VIGNETO), resulting in 384 genotyped varieties using the 18k SNP genotyping array. Varieties belonging to Eurasia, from the Caucasus to Western Europe (De Lorenzis et al., 2015, 2019; Laucou et al., 2018), were added for the final dataset of 1,038 unique SNP-profiles.

### History of the Italian Population and Distinctness From the Other Eurasian Populations

The origin of Italian viticulture dates to the Etruscans (around the 8^th^ BC) and Greeks (7^th^-6^th^ BC). The former domesticated wild grapevines in ITAP-north-center, while the Greeks introduced their varieties in ITAP-south. Differences between these areas occurred in winegrowing, the choice of varieties, and in the way to manage the vines (Buono and Vallariello, 2002), but these differences were not highlighted yet by genetic analysis. Furthermore, the Italian varieties did not appear distinguishable from those of the other European regions. The first research including a considerable panel of Italian varieties, genotyped at 34 SSR loci, was unable to cluster the germplasm based on their geographic origin (Cipriani et al., 2010). Later, 304 Italian varieties, included in a large germplasm collection from the Information System of the National Institute for Agronomical Research (INRA), were characterized by using another panel of 20 SSR loci, resulting admixed by a weak genetic structure based on geographic origin (Bacilieri et al., 2013). Recently, 783 Eurasian varieties belonging to four grapevine repositories were characterized with the 10k genome-wide SNPs and the 84 Italian cultivars clustered together with WCEUR genotypes, confirming the indiscernibility of the Italian germplasm (Laucou et al., 2018). Then, 140 varieties from ITAP-south characterized by the same 18k SNPs showed similarity with genotypes coming from both Eastern Mediterranean Sea and France, although the ITAP-south cluster appeared evident (De Lorenzis et al., 2019). D'Onofrio et al. (2021) highlighted that most of the Italian varieties, genotyped by SNPs, appeared structured in few main clusters with the first and second-degree relationship scattered from the South to North of Italy.

Here, for the first time, the cluster analysis on 384 Italian genotypes allowed us to distinguish the Northern and Southern genotypes, appearing clearly separated into two large clusters by using both UPGMA algorithm (Figure 1A) and PCoA (Supplementary Figure 1). The observed distinctiveness was better detailed by using a multiple Bayesian approach, DAPC (Figures 1B,C and Supplementary Table 1). A significant *K* = 3 was evidenced with a group (Pool 2) including South-Italian varieties and two others overlapped, the first mainly counting North-Italian varieties (Pool 1), and the second (Pool 3) including varieties from all Italian areas (Figure 1B). In each pool, some ancestors can be recognized, such as Garganega/Grecanico dorato in Pool 1, Bombino and Visparola in Pool 3 (D'Onofrio et al., 2021) and Mantonico bianco and Sangiovese in Pool 2 (De Lorenzis et al., 2019; D'Onofrio et al., 2021) (Supplementary Table 1). These last could represent “bridge varieties” linking ITAP-south and ITAP-north-center populations.

The complex origin of ITAP-south germplasm was confirmed; indeed the pairwise fixation index, *Fst* was the same in both couples ITAP-south/BALK and ITAP-south/ITAP-north-center (Supplementary Table 5 and Figure 3A). The higher proximity of ITAP-south to BALK than to ITAP-north-center germplasm was inferred by other analyses, such as MDS with IBD, NJ tree, and TreeMix (Figures 3B,D, 4). Otherwise, ITAP-north-center varieties showed a higher proximity to WCEUR germplasm (Figures 3A–C, 4C). The *Fst* in the couple ITAP-north-center/WCEUR was the lowest among all pairwise comparisons (Supplementary Table 5). Moreover, one major migration event from Southern to Northern Italy, inferred with TreeMix analysis (Figure 4C), is supported by a recent pedigree study (D'Onofrio et al., 2021) and the possible “bridge” role of the varieties included in Pool 3 (DAPC) (Figures 1B,C and Supplementary Table 1). In addition, Scenario 1 (ABC analysis) agrees with this hypothesis, as the best supported scenario for a mixed origin of ITAP-north-center germplasm between ITAP-south and WCEUR genotypes (Figure 6B). The ITAP-north-center and ITAP-south genotypes were distinguished, also when compared to the Eurasian dataset. Indeed, the DAPC on the whole SNP dataset (1,038 genotypes) split the ITAP germplasm in two groups (Figures 2B,C and Supplementary Table 1). Following the posterior membership probabilities (>70%), Pools 6 and 7 included mainly varieties from WCEUR and ITAP-north/center, respectively (Supplementary Table 1). Once again, ITAP-north and ITAP-south varieties were distinguishable, while ITAP-center germplasm was split. Small groups of Italian varieties were pinpointed in other pools, e.g., some Sardinian cultivars, historically racked from Spain to Sardinia (De Mattia et al., 2009), grouped in Pool 8, representative of the IBER germplasm. Some muscat flavored varieties grouped in Pool 5, where the offspring of the well-recognized major ancestor Moscato bianco/Muscat à petits grains blanc were placed (Cipriani et al., 2010; Ruffa et al., 2016). Finally, the geographic location can account for some cultivars from the Adriatic coast grouped into Pool 2, including BALK germplasm.

A slightly more complex picture was defined by ancestry coefficient analysis (R/TESS3) on the whole dataset, where ITAP-south germplasm resulted as the main contributor to *K5*, while ITAP-north-center cultivars showed the highest values of membership in two pools, *K4* and *K7*, with *K7* appearing as a mixed pool (Figure 5 and Supplementary Table 8).

### Genetic Structure of the Eurasian Germplasm

The genetic distances among the six Eurasian populations (Figure 3A) indicated the highest divergence between EMCA-MFEAS-RUUK and WCEUR populations, which was also confirmed by the highest *Fst* index among all pairwise comparisons (Supplementary Table 5). This result was expected given the well-known gene flow from Eastern to Western Eurasia. Our results appeared in agreement with an evoked divergence among the grapevine genetic structures in different European grapevine-growing regions, firstly reported by Sefc et al. (2000). Bayesian computations helped us depict a more detailed reconstruction of this history.

The DAPC performed on the whole dataset highlighted nine pools, attributable to the Eurasian populations (Figures 2B,C). Seven out of nine pools appeared correlated with the main geographic memberships (Supplementary Table 1). Indeed, Pools 6 and 7 included nearly all Italian varieties, Pool 8 included the highest number of varieties from IBER, Pools 3 and 9 represented the EMCA-MFEAS-RUUK germplasm; noticeably, the Georgian varieties were grouped in Pool 9, while Pools 1 and 4 included many varieties from WCEUR population. Finally, Pool 2 comprised many varieties of mixed geographic origin, two main parents, Gouais blanc/Heunischweiss and Blank Blauer/Vulpea, ascribed to WCEUR, but also a predominance of BALK varieties. The geographic membership of Pool 2 varieties is still questioned (Bowers et al., 1999; Crespan et al., 2020), and our results suggested their potential “bridge” role. Interestingly, Pool 5 resulted mixed between IBER and WCEUR germplasm putatively explaining the migration event from IBER to WCEUR population, highlighted by TreeMix analysis (Figure 4C) and supported in ABC results on Scenario 7 (Figure 6C). This appears to be a new finding in contrast with previous reports that hypothesized a migration of some cultivars from WCEUR to IBER population after a secondary domestication event in IBER (Arroyo-García et al., 2006).

The ancestry coefficient analysis highlighted one less genetic pool than DAPC (*K* = *8*) of which seven pools showed the highest ancestry membership values in agreement with the geographic origin. Similarly, Pools from 1 to 5 were assigned to IBER, EMCA-MFEAS-RUUK, Georgian, ITAP-north-center, and ITAP-south populations, respectively (Figure 5B and Supplementary Table 8). Pool 6 was mainly shared by the BALK varieties, deserving additional comments: three varieties from Cyprus harbor a very low ancestry and should have been ascribed to EMCA-MFEAS-RUUK. Notably, the Greek varieties showed multiple memberships, with similar ancestry values for *K2* (including EMCA-MFEAS-RUUK germplasm) and *K6* pools. Therefore, Greece appears to be the main bridge between Near Eastern germplasm and the Mediterranean Basin (ITAP), supporting the theory that grapevines were spread from the Greek shores to the Southern and then Northern Italian ones (Buono and Vallariello, 2002). The *K7* pool resulted largely admixed, in which Swiss varieties showed the highest ancestry value, followed by ITAP-north-center. The *K8* pool encompassed the WCEUR germplasm. Unexpectedly, the Portuguese varieties showed a greatest membership to *K8* (Supplementary Table 8), higher than *K1* (IBER pool), appearing to be the most plausible candidate group for the genetic exchange between IBER and WCEUR, highlighted by TreeMix computations and DAPC analysis. This hypothesis is also supported by ancestry coefficient analysis, where Portugal is painted with two colors (Figure 5A).

In summary, IBER germplasm appeared distinguished from the others indicating a complex history supported by genetic relationship (Figure 2 and Supplementary Figure 4), IBD, and NJ analyses (Figures 3B–D). The genetic structure analysis stated that IBER varieties included two groups, one related to WCEUR group and the other to Northern Africa and Eastern varieties (Bacilieri et al., 2013). Through structure analysis, ten IBER varieties (Supplementary Table 1) showed a larger ancestry membership to Pool 3 (EMCA-MFEAS-RUUK) than pool 8 (IBER). They could have been derived from the migration event from EMCA-MFEAS-RUUK to IBER, highlighted by TreeMix analysis (Figure 4C) and mediated by MAGH, as hypothesized in Scenario 10 by ABC analysis (Figure 6D). The groups defined by DAPC and the ancestry coefficient analyses sustained a major migration event from IBER to WCEUR population (TreeMix), explained by Portuguese varieties falling in Pool 4 (WCEUR) (Supplementary Table 1), Pool 5 (WCEUR and IBER) (Supplementary Table 4) and *K8* (Supplementary Table 8), respectively. Moreover, the Scenario 7 from ABC analysis well-supported a gene flow from ITAP-south to IBER and then to WCEUR population (Figure 6C), as hypothesized by Buono and Vallariello (2002).

### LD-Values Differed in the Six Geographic Groups

Standard *r*^2^-value used to perform LD measure is a parameter suffering from bias when the individuals are dependent, or the analyzed populations evolved differently. In such cases, long-range values for LD are obtained as admixed populations (Mangin et al., 2012). Therefore, the distinctiveness of the evolution of six grape populations in their different geographic contexts, leading to differences in the allele frequencies affecting *r*^2^-values appeared indubitable. Accordingly, shorter LD-values can account for a higher number of meiotic events and recombination within the population, leading to limited relationships. By contrast, high levels of admixture, close relationships, and low recombination rates, lead to longer LD-values (Mangin et al., 2012).

Providing that these observations are not significant on a time scale; according to LD trend, EMCA-MFEAS-RUUK population showed a more natural and faster evolution, followed by BALK and ITAP germplasm, while IBER and WCEUR varieties appeared more static and closely related (Figure 3C). The admixture within WCEUR and IBER populations, and the gene flow from WCEUR to IBER germplasm, were already established by pedigree analyses (Bowers et al., 1999; Boursiquot et al., 2009; Diaz Losada et al., 2010; Zinelabidine et al., 2015). Moreover, archaeological research in France highlighted that vines remain closely related to the Western European cultivars used nowadays for winemaking. The varieties cultivated by the Romans in ancient times were the same as those grown today or shown to be parent-offspring related with present varieties (Ramos-Madrigal et al., 2019).

### The East-to-West Grapevine Gene Flow

Grapevine domestication seems to be related to winemaking, although which process predated the other remains questionable (Terral et al., 2010). The pivotal role of Greece in the viticulture history is well-known. Indeed, the Greeks achieved mastery in grapevine cultivation and winemaking, elevating both to a cultural phenomenon. There is no reliable information on the introduction of winemaking techniques in Greece. From Greece, the culture of wine reached Western Europe through Southern Italy and then France and Spain (Buono and Vallariello, 2002; Scienza, 2004). This migration flow from the East to West also involved grapevine cultivars, recently documented by genetic analysis (Myles et al., 2011; De Lorenzis et al., 2019; D'Onofrio et al., 2021). Our results confirmed this East-to-West gene flow. The population pairwise *Fst* estimation assigned the lowest genetic distance to ITAP-north-center and WCEUR pair, while the highest was found between WCEUR and EMCA-MFEAS-RUUK (Supplementary Table 5). The scaling of NJ-tree branches related to WCEUR population reflected a more recent development (Figure 3A). What has happened in between has to be analyzed. The complex role played by Italian germplasm evoked in this work, suggested a key breakthrough to understanding the grapevine germplasm evolution in Eurasia. Genetic data analysis (Figures 3A, 4) confirmed gene flow events from EMCA-MFEAS-RUUK to BALK and then to ITAP-south population. The novelty of the findings is the relationship between ITAP-north-center and WCEUR populations (Figures 3A–C, 4). The ABC analysis suggested that the admixture of ITAP-north-center germplasm could be derived by the gene flow from both ITAP-south and WCEUR varieties (Figure 6B, Scenario 6).

The gene flow from ITAP-south to WCEUR throughout IBER population assigned an additional role to the *Magna Graecia* (Figures 4C, 6C, Scenario 7). The complex structure of IBER population is also affected by the additional gene flow from EMCA-MFEAS-RUUK through the MAGH population, as highlighted by gene flow, ancestry evaluation, and ABC analysis through Scenario 10 (Figures 4, 5, 6D). As expected, the WCEUR population was the last member of this chain, showing the highest distance from the EMCA-MFEAS-RUUK group. In turn, WCEUR varieties played a role in gene flow, receiving a contribution from IBER, and contributing to the ITAP-north-center germplasm (Figures 4, 6, Scenario 6).

After the first grapevine domestication event in the Caucasus, cultivated vines spread southwards to Anatolia and Egypt 5,000 years ago, as well as around the Mediterranean Basin, following the main civilizations (McGovern, 2003; This et al., 2006). In this scenario, the key role of Italian germplasm in the grape genetic differentiation (Grassi et al., 2003; Sunseri et al., 2018; De Lorenzis et al., 2019) was confirmed. We provide a sophisticated genetic model that is useful to ultimately attribute the role of frontier between the Western and Eastern Eurasia to the Italian and mainly to the *Magna Graecia* germplasm.

Our finding, reconciling genetic and archaeological data for one of the most cultivated and fascinating crops in the world, has made a key contribution to defining the genetic relationships among grapevine populations distributed in a wide geographic area ranging from the Caucasus to the Iberian Peninsula.

## Data Availability Statement

The datasets presented in this study can be found in online repositories. The names of the repository/repositories and accession number(s) can be found in the article/supplementary material.

## Author Contributions

FM, GDL, MC, and FS: conceptualization. FM and GDL: methodology, formal analysis, and writing—original draft. AM, MZ, CM, PR, LB, CD'O, MB, CB, LP, and VN: investigation. CM, PR, LB, CD'O, MB, CB, LP, and VN: resources. FM, MC, and FS: supervision. CM, PR, LB, CD'O, MB, CB, LP, FS, and VN: funding acquisition. All authors contributed to the writing—review and editing, article, and approved the submitted version.

## Conflict of Interest

The authors declare that the research was conducted in the absence of any commercial or financial relationships that could be construed as a potential conflict of interest.

## Publisher's Note

All claims expressed in this article are solely those of the authors and do not necessarily represent those of their affiliated organizations, or those of the publisher, the editors and the reviewers. Any product that may be evaluated in this article, or claim that may be made by its manufacturer, is not guaranteed or endorsed by the publisher.
