# Supplementary material for: Integrated Bayesian Approaches Shed Light on the Dissemination Routes of the Eurasian Grapevine Germplasm
Source: Front Plant Sci. 2021 Aug 5;12:692661. doi: 10.3389/fpls.2021.692661 (PMC8381769; doi:10.3389/fpls.2021.692661)
Supplement: Supplementary file 2 [file Data_Sheet_1.docx]

Supplementary Material

# **Supplementary Tables**

**
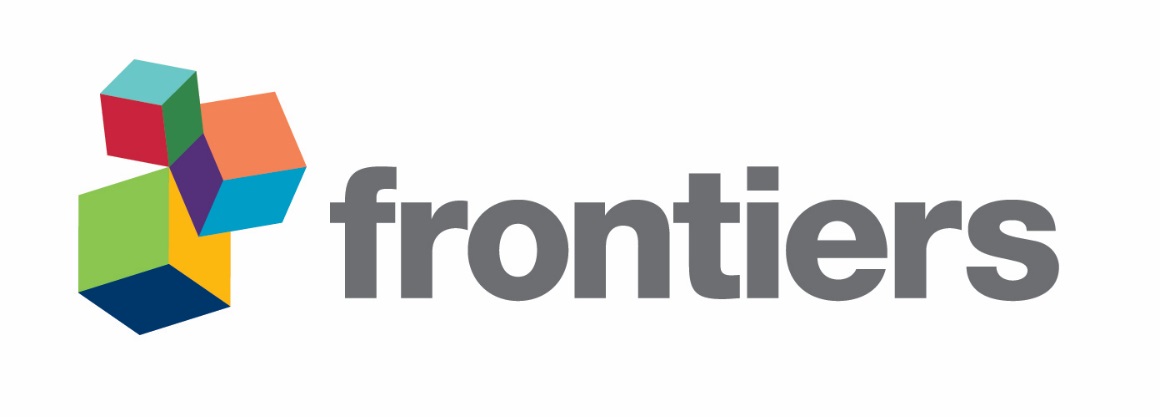
**

**Supplementary Table S1** List of grapevine varieties. Name, geographic origin, membership collection, SNP profiles, ancestry and probability of posterior membership for each sample included in the present study were reported. All varieties were genotyped using the GrapeReSeq 18K Vitis (Le Paslier et al. 2013). Raw data was processed using a robust filtering: markers showing call quality values (p50GC) lower than 0.54 were removed, as well as loci having GenTrain (GT) score values lower than 0.6 (De Lorenzis et al. 2015), missingness rate > 1% and a minor allele frequency (MAF) < 5%. Afterward, individual missingness was calculated and varieties with more than 5% of missing values were excluded. Finally, the duplicated profiles detected by calculating the pairwise percentage of mismatches between individuals were deleted. Italian genotypes were gathered into their passport data, Northern, Central and Southern Italy, all the other genotypes were divided into 4 groups: i) Iberian Peninsula (IBER), ii) Western and Central Europe (WCEUR), iii) Balkans (BALK), iv) Russia, Ukraine, Eastern Mediterranean, Caucasus, Middle and Far East (EMCA-MFEAS-RUUK) (Table 1), following the previous geographical assignments (Laucou et al., 2018).

This table has been uploaded as separate excel file (Table_S1_Mercati_et_al_fpls).

**Supplementary Table S2** BIC values (from *K* =1 to *K* = 20) for both the Italian germplasm (384 varieties) and the whole grapevine collection (1,038 accessions) studied. In bold the model chosen (*K*) with the lowest BIC for each panel.

| **K** | **BIC**  **(Italian**  **germplasm)** | **BIC**  **(wide grapevine collection)** |
| --- | --- | --- |
| 1 | 2854.950 | 7956.437 |
| 2 | 2844.163 | 7913.572 |
| **3** | **2843.307** | 7899.882 |
| 4 | 2843.868 | 7889.497 |
| 5 | 2844.994 | 7882.009 |
| 6 | 2846.209 | 7879.188 |
| 7 | 2847.778 | 7872.426 |
| 8 | 2850.219 | 7869.806 |
| **9** | 2851.566 | **7867.521** |
| 10 | 2854.338 | 7867.560 |
| 11 | 2856.524 | 7867.703 |
| 12 | 2859.213 | 7868.302 |
| 13 | 2860.236 | 7869.854 |
| 14 | 2864.357 | 7872.15 |
| 15 | 2867.102 | 7873.129 |
| 16 | 2869.150 | 7875.548 |
| 17 | 2873.561 | 7877.438 |
| 18 | 2875.619 | 7879.986 |
| 19 | 2878.284 | 7881.868 |
| 20 | 2882.118 | 7883.055 |

**Supplementary Table S3** Percentage of varieties belonging to Italian germplasm classified at the best *K* (3) showing the ancestry membership and the posterior membership probabilities (>70%), by using fastStructure and DAPC analysis, respectively. The samples are grouped by origin (ITAP-north, ITAP-center, and ITAP-south).

| **Group** | **N.** | **Ancestry membership > 70% (fastStructure)** | | | |
| --- | --- | --- | --- | --- | --- |
|  |  | **N_m_** | **Pool 1** | **Pool 2** | **Pool 3** |
| ITAP-north | *143* | 71 (49%) | 62 (87%) | - | 9 (13%) |
| ITAP-center | *105* | 51 (48%) | 28 (55%) | 4 (8%) | 19 (37%) |
| ITAP-south | *136* | 74 (54%) | 3 (4%) | 30 (41%) | 41 (55%) |
| *TOT* | *384* | *196* | *93* | *34* | *69* |
| **Group** | **N** | **Posterior membership probabilities > 70% (DAPC)** | | | |
|  |  | **N_m_** | **Pool 1** | **Pool 2** | **Pool 3** |
| ITAP-north | *143* | 141 (99%) | 116 (82%) | - | 25 (18%) |
| ITAP-center | *105* | 102 (97%) | 51 (50%) | 5 (5%) | 46 (45%) |
| ITAP-south | *136* | 135 (99%) | 6 (5%) | 42 (31%) | 87 (64%) |
| *TOT* | *384* | *378* | *173* | *47* | *158* |

*N. = total number of samples; N_m_ = number of samples with ancestry membership and a posterior membership probability > 70%*

**Supplementary Table S4** Mean of ancestry membership (fastStructure) and posterior membership probabilities (DAPC) evaluated at the best *K* (9) for each group (Table 1) belonging to the wide grapevine germplasm (1,038 varieties). As reported in Table 1, the samples were gathered in six groups based on their geographic origin. In bold the more representative pools for each group (>20%).

| **Group** | **Mean of the ancestry membership (fastStructure)** | | | | | | | | |
| --- | --- | --- | --- | --- | --- | --- | --- | --- | --- |
|  | **Pool 1** | **Pool 2** | **Pool 3** | **Pool 4** | **Pool 5** | **Pool 6** | **Pool 7** | **Pool 8** | **Pool 9** |
| BALK | 0.00581 | **0.41847** | **0.24906** | 0.01742 | 0.04048 | 0.13852 | 0.01442 | 0.11147 | 0.00436 |
| EMCA-MFEAS-RUUK | 0.02830 | 0.06254 | **0.53597** | 0.00078 | 0.01162 | 0.03526 | 0.00215 | 0.02553 | **0.29786** |
| IBER | 0.08789 | 0.02119 | 0.10779 | 0.12105 | 0.02210 | 0.03491 | 0.01293 | **0.58974** | 0.00240 |
| ITAP-north-center | 0.11680 | 0.07957 | 0.04014 | 0.04388 | 0.08097 | 0.17724 | **0.41541** | 0.04046 | 0.00554 |
| ITAP-south | 0.05739 | 0.05437 | 0.12742 | 0.01086 | 0.05034 | **0.5472**3 | 0.04536 | 0.10288 | 0.00415 |
| WCEUR | **0.24201** | 0.13950 | 0.04600 | **0.25804** | 0.06093 | 0.05725 | 0.06851 | 0.12545 | 0.00231 |
| **Group** | **Mean of posterior membership probabilities (DAPC)** | | | | | | | | |
|  | **Pool 1** | **Pool 2** | **Pool 3** | **Pool 4** | **Pool 5** | **Pool 6** | **Pool 7** | **Pool 8** | **Pool 9** |
| BALK | 0.01600 | **0.60526** | **0.20066** | 0.00768 | 0.11124 | 0.05111 | 0.00804 | 5.12E-09 | 2.65E-31 |
| EMCA-MFEAS-RUUK | 1E-10 | 0.08223 | **0.58216** | 0.00629 | 0.02353 | 0.00043 | 4.8E-05 | 5.46E-12 | **0.30531** |
| IBER | 0.10880 | 0.00527 | 0.02880 | 0.08742 | **0.32758** | 6.07E-06 | 0.0091 | **0.43298** | 3.93E-34 |
| ITAP-north-center | 0.01190 | 0.0872 | 8.48E-07 | 0.03733 | 0.02589 | **0.24361** | **0.59045** | 0.00364 | 2.85E-34 |
| ITAP-south | 2.74E-09 | 0.05867 | 0.0511 | 4.02E-05 | 0.09254 | **0.73197** | 0.03622 | 0.02942 | 1.98E-35 |
| WCEUR | **0.25231** | 0.07255 | 0.01841 | **0.36154** | **0.21596** | 0.01694 | 0.0561 | 0.00622 | 2.2E-29 |

**Supplementary Table S5** Population pairwise fixation index (*Fst*) among the grapevine groups (Supplementary Table S1).

|  | **BALK** | **EMCA-MFEAS-RUUK** | **IBER** | **ITAP-north-center** | **ITAP-south** | **WCEUR** |
| --- | --- | --- | --- | --- | --- | --- |
| BALK | - |  |  |  |  |  |
| EMCA-MFEAS-RUUK | 0.013 | - |  |  |  |  |
| IBER | 0.018 | 0.024 | - |  |  |  |
| ITAP-north-center | 0.018 | 0.024 | 0.021 | - |  |  |
| ITAP-south | 0.012 | 0.019 | 0.017 | 0.012 | - |  |
| WCEUR | 0.025 | 0.033 | 0.021 | 0.009 | 0.021 | - |

**Supplementary Table S6** The optimal number of migrations edges evaluated through an *ad hoc* statistic based on the Evanno’s method. The columns in the table are: "m" - number of migration edges; "runs" = number of iterations for "m"; " Lm (mean)" - mean log likelihood across runs; "sd (Lm)" - standard deviation of log likelihood across runs; "Lm (min)" - minimum log likelihood across runs; "Lm (max)" - maximum log likelihood across runs;"*Δm*" - the ad hoc delta M statistic (second-order rate of change in log likelihood); "f (mean)" -mean proportion of variation explained by the models; "sd (f)" - standard deviation of the proportion of variation explained by the models.

| **m** | **runs** | **Lm**  **(mean)** | **sd**  **(Lm)** | **Lm**  **(min)** | **Lm**  **(max)** | ***Δm*** | **f**  **(mean)** | **sd**  **(f)** |
| --- | --- | --- | --- | --- | --- | --- | --- | --- |
| 0 | 50 | -350.502 | 34.435 | -438.953 | -283.580 | NA | NA | NA |
| 1 | 10 | 17.760 | 4.545 | 13.539 | 25.192 | 61.064 | 0.979 | 0.002 |
| 2 | 10 | 108.467 | 0.591 | 107.816 | 109.099 | 75.212 | 0.992 | 0.001 |
| 3 | 10 | 154.744 | 7.832 | 145.813 | 166.242 | 4.244 | 0.997 | 0.000 |
| 4 | 11 | 167.783 | 5.750 | 153.856 | 177.987 | 0.708 | 0.998 | 0.001 |
| 5 | 9 | 176.754 | 2.166 | 174.068 | 179.271 | NA | 0.999 | 0.001 |

**Supplementary Table S7** Number of times for directional migration events inferred in 10 TreeMix runs at *m* = 4. Only the events with high significance levels (*p* < 0.001) were showed.

| **Migration events at *m* = 4 (from / to)** | **Number of times**  **(*p* < 0.001)** |
| --- | --- |
| EMCA-MFEAS-RUUK / IBER | 7 |
| ITAP-south / ITAP-north-center | 6 |
| ITAP-north-center / EMCA-MFEAS-RUUK | 6 |
| IBER / WCEUR | 5 |
| WCEUR / ITAP-north-center | 5 |
| EMCA-MFEAS-RUUK / ITAP-north-center | 2 |
| BALK / ITAP-south | 1 |

**Supplementary Table S8** Ancestry value evaluated for each country included in the R/TESS3 analysis. The number of samples (N) and coordinates used for each country were reported. Each *K* was highlighted using the color reported in Fig. 5.

This table has been uploaded as separate excel file (Table_S8_Mercati_et_al_fpls).

**Supplementary Table S9** Summary statistics of simulated and observed datasets for ABC analysis displayed in Figure 6. The most likely scenarios were in bold and italic.

| Summary statistics ^a^ | Observed values | ***Scenario 1*** |  | Scenario 2 |  | Scenario 3 |  |
| --- | --- | --- | --- | --- | --- | --- | --- |
| HMO_1 | 0.2866 | ***0.9783*** | (**) | 0.9711 | (**) | 0.9702 | (**) |
| HMO_2 | 0.2906 | ***0.9850*** | (**) | 0.9897 | (**) | 0.9879 | (**) |
| HMO_3 | 0.2982 | ***0.8982*** | (*) | 0.9954 | (**) | 0.9956 | (**) |
| FMO_1&2 | 0.0180 | ***0.0232*** |  | 0.0080 |  | 0.0090 |  |
| FMO_1&3 | 0.0317 | ***0.0288*** |  | 0.0007 | (**) | 0.0007 | (*) |
| FMO_2&3 | 0.0201 | ***0.0212*** |  | 0.0424 |  | 0.0437 |  |
| NMO_1&2 | 0.0105 | ***0.0273*** |  | 0.0001 | (**) | 0.0001 | (*) |
| NMO_1&3 | 0.0161 | ***0.0087*** |  | 0.0005 | (**) | 0.0075 |  |
| NMO_2&3 | 0.0110 | ***0.0321*** |  | 0.0375 |  | 0.0384 |  |
| Summary statistics | Observed values | Scenario 4 |  | Scenario 5 |  | ***Scenario 6*** |  |
| HMO_1 | 0.2868 | 0.9751 | (**) | 0.9760 | (*) | ***0.9826*** | (*) |
| HMO_2 | 0.2970 | 0.9989 | (**) | 0.9988 | (**) | ***0.9907*** | (**) |
| HMO_3 | 0.2923 | 0.9972 | (**) | 0.9968 | (*) | ***0.9977*** | (**) |
| FMO_1&2 | 0.0197 | 0.0102 |  | 0.0131 |  | ***0.0135*** |  |
| FMO_1&3 | 0.0274 | 0.0187 |  | 0.0215 |  | ***0.0206*** |  |
| FMO_2&3 | 0.0151 | 0.0307 |  | 0.0303 | (**) | ***0.0322*** |  |
| NMO_1&2 | 0.0108 | 0.0132 |  | 0.0101 |  | ***0.0146*** |  |
| NMO_1&3 | 0.0149 | 0.0104 |  | 0.0111 |  | ***0.0095*** |  |
| NMO_2&3 | 0.0098 | 0.031 | (**) | 0.0306 |  | ***0.0341*** |  |
| Summary statistics | Observed values | ***Scenario 7*** |  | Scenario 8 |  | Scenario 9 |  |
| HMO_1 | 0.2883 | ***0.9737*** | (*) | 0.9808 | (*) | 0.9896 | (*) |
| HMO_2 | 0.2928 | ***0.9954*** | (**) | 0.9974 | (**) | 0.9938 | (**) |
| HMO_3 | 0.2926 | ***0.8959*** | (**) | 0.8821 | (**) | 0.9818 | (**) |
| FMO_1&2 | 0.0204 | ***0.0202*** |  | 0.0212 |  | 0.0439 |  |
| FMO_1&3 | 0.0272 | ***0.0174*** |  | 0.0173 |  | 0.0124 |  |
| FMO_2&3 | 0.0190 | ***0.0437*** |  | 0.0236 |  | 0.0242 |  |
| NMO_1&2 | 0.0115 | ***0.0221*** |  | 0.0131 |  | 0.0407 | (*) |
| NMO_1&3 | 0.0149 | ***0.0163*** |  | 0.0193 |  | 0.0254 |  |
| NMO_2&3 | 0.0119 | ***0.0423*** |  | 0.0262 | (*) | 0.0262 |  |
| Summary statistics | Observed values | ***Scenario 10*** |  | Scenario 11 |  | Scenario 12 |  |
| HMO_1 | 0.2904 | ***0.9809*** | (*) | 0.9870 | (*) | 0.9955 | (*) |
| HMO_2 | 0.3131 | ***1.0000*** | (*) | 1.0000 | (*) | 1.0000 | (*) |
| HMO_3 | 0.2884 | ***0.9943*** | (**) | 0.9762 | (*) | 0.9781 | (*) |
| FMO_1&2 | 0.1581 | ***0.2840*** |  | 0.0060 | (**) | 0.4944 |  |
| FMO_1&3 | 0.0218 | ***0.0003*** | (**) | 0.0036 | (**) | 0.0002 | (**) |
| FMO_2&3 | 0.1609 | ***0.3034*** |  | 0.0023 | (**) | 0.2472 |  |
| NMO_1&2 | 0.1167 | ***0.1081*** |  | 0.1087 |  | 0.3264 |  |
| NMO_1&3 | 0.0134 | ***0.0182*** |  | 0.0061 | (**) | 0.0001 | (**) |
| NMO_2&3 | 0.1184 | ***0.6338*** |  | 0.4006 |  | 0.3145 |  |

*^a^ HMO: Genic diversity, mean of complete distribution; FMO: FST distances, mean of complete distribution; NMO; Nei’s distances, mean of complete distribution; 1, 2, 3: populations; *: p > 0.05; **: p > 0.01.*

**Supplementary Table S10** Performance evaluation for the ABC analysis displayed in Fig. 6. The most likely scenarios were in bold and italic.

| **Performance evaluation** | | | |
| --- | --- | --- | --- |
| **Hypothesis 1** |  |  |  |
| ABC performance | ***Scenario 1*** | Scenario 2 | Scenario 3 |
| D1 | ***77.3*** | 17.1 | 8.4 |
| D2 | ***12.6*** | 64.2 | 22.4 |
| D3 | ***3.5*** | 3.2 | 56.1 |
| Type I error (mean) | ***14.0*** | 13.5 | 10.8 |
| Type II error (mean) | ***10.3*** | 11.4 | 7.3 |
| **Hypothesis 2** |  |  |  |
| ABC performance | Scenario 4 | Scenario 5 | ***Scenario 6*** |
| D4 | 67.3 | 24.5 | ***8.2*** |
| D5 | 21.2 | 69.4 | ***9.4*** |
| D6 | 5.2 | 7.6 | ***87.2*** |
| Type I error (mean) | 13.2 | 16.1 | ***8.8*** |
| Type II error (mean) | 16.3 | 15.3 | ***6.4*** |
| **Hypothesis 3** |  |  |  |
| ABC performance | ***Scenario 7*** | Scenario 8 | Scenario 9 |
| D7 | ***92.2*** | 7.2 | 0.6 |
| D8 | ***9.2*** | 89.8 | 1 |
| D9 | ***22.2*** | 25.4 | 52.4 |
| Type I error (mean) | ***15.7*** | 16.3 | 0.8 |
| Type II error (mean) | ***3.9*** | 5.1 | 23.8 |
| **Hypothesis 4** |  |  |  |
| ABC performance | ***Scenario 10*** | Scenario 11 | Scenario 12 |
| D10 | ***88.0*** | 10.8 | 1.2 |
| D11 | ***11.8*** | 87.8 | 0.4 |
| D12 | ***25.3*** | 27.5 | 47.2 |
| Type I error (mean) | ***18.6*** | 19.2 | 0.8 |
| Type II error (mean) | ***6.0*** | 6.1 | 26.4 |

*D: Number of times the scenario has the highest posterior probability.*

# **Supplementary Figures**

**
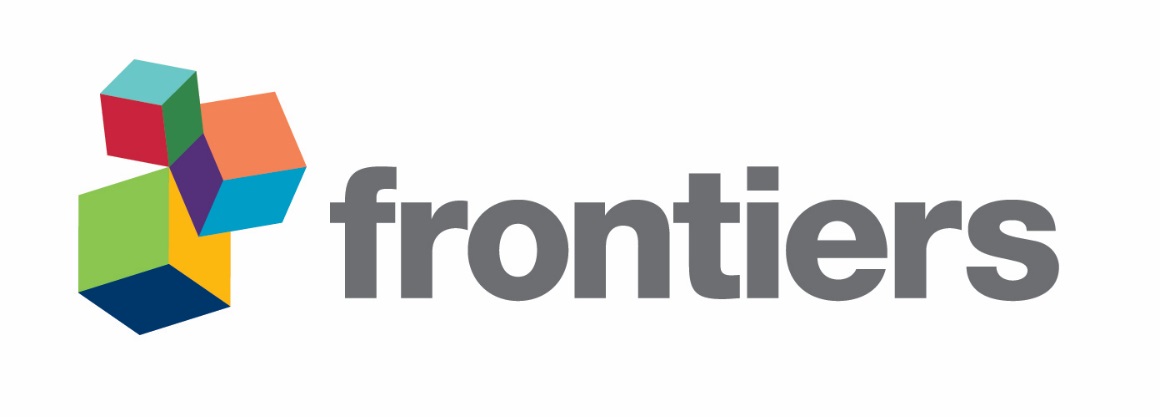
**

**
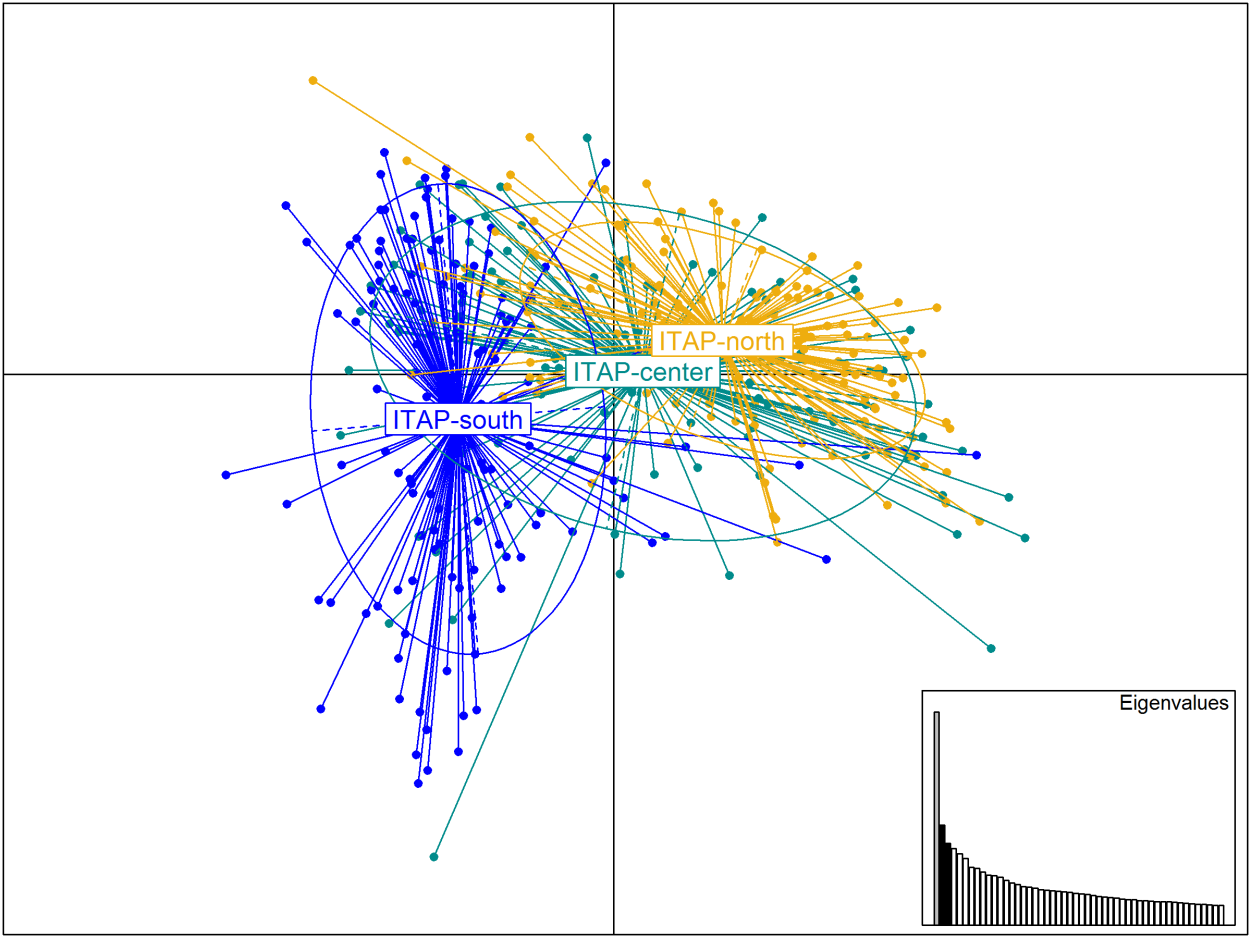
**

**Supplementary Figure S1** Principal coordinates analysis (PCoA) of Italian germplasm. The varieties were highlighted based on their geographical origin: Northern Italy (ITAP-north), light brown; Central Italy (ITAP-center), cyan; Southern Italy (ITAP-south), blue.

**Supplementary**
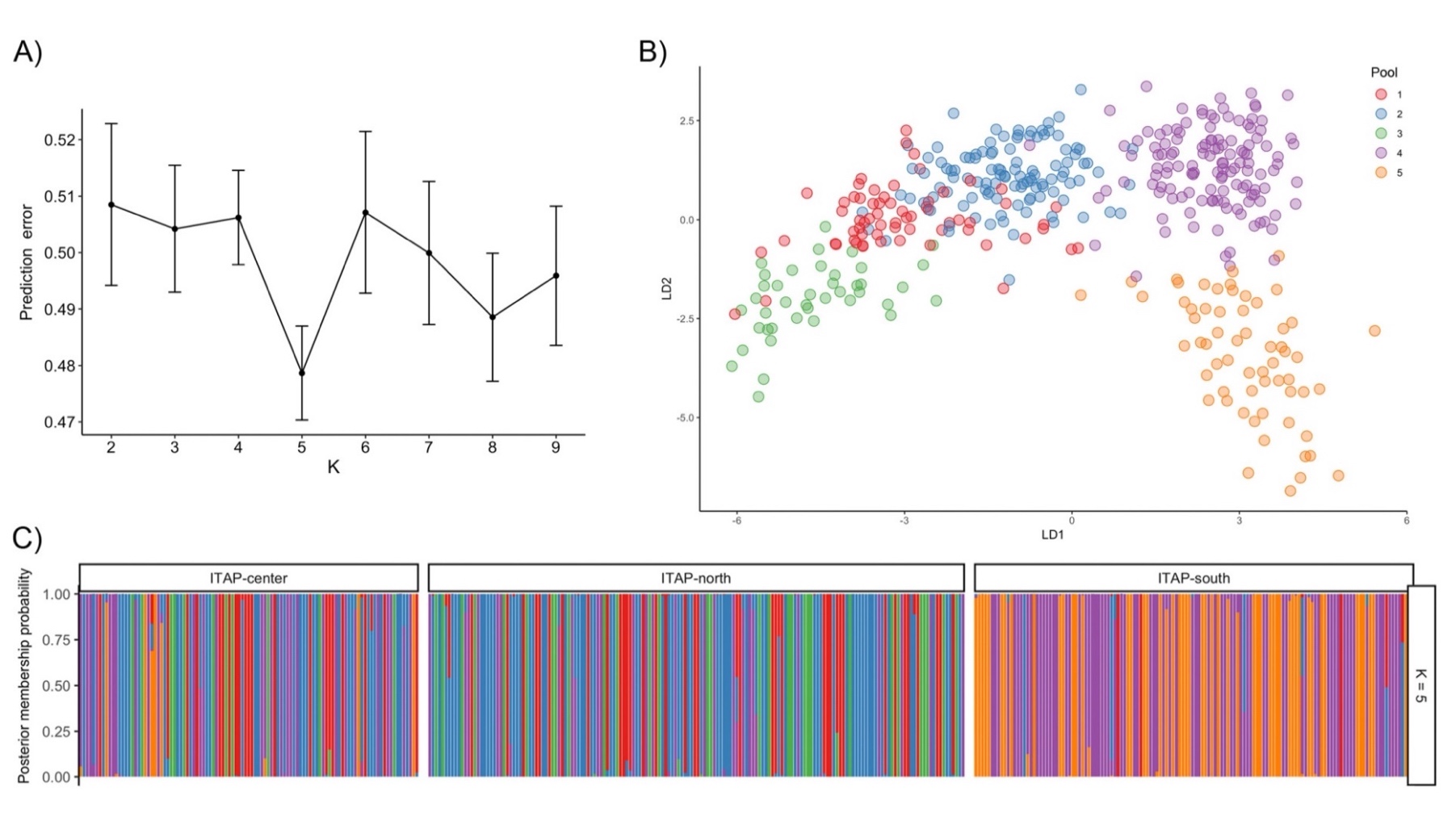
**Figure S2** **A)** *K* values ranging from 1 to 9 were tested through fastStructure for 384 Italian grapevine varieties. The fitting number of model complexity explaining the structure of the datasets investigated was chosen applying the algorithm for multiple choices *chooseK.py*, highlighting that the best *K* value was included between 2 and 6 (Model complexity that maximizes marginal likelihood = 6; Model components used to explain structure in data = 2). To extract the optimum *K*, the prediction error for each *K* was computed by the cross validation (*--cv*) function. The lowest model complexity (*K*=5) above which prediction errors do not vary significantly is considered the best one. **B)** DAPC scatter plot based on discriminant functions of the best *K* (5) obtained from cross-validation (prediction error). **C)** Barplot of the posterior probability for *K* = 5 were developed to easily identify the genotypes belonging to the different genetic pools found. The varieties were highlighted based on their geographical origin (ITAP-center, ITAP-north, and ITAP-south).


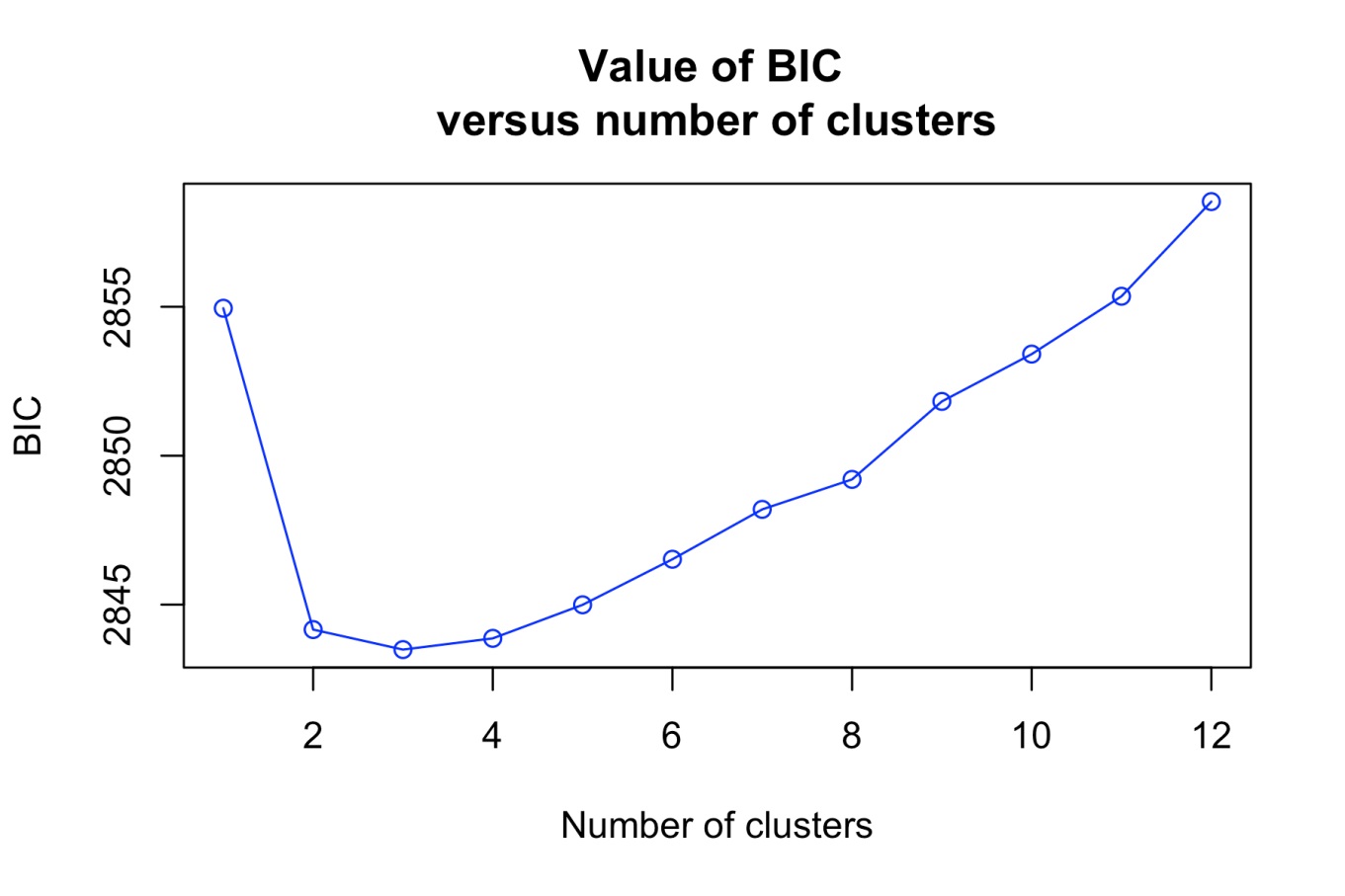


**Supplementary Figure S3** Bayesian Information Criterion (BIC) evaluated through Discriminant Analysis of Principal Components (DAPC). BIC approach was used to infer the *K*-means clustering within the Italian grapevines (*K*=3).

**
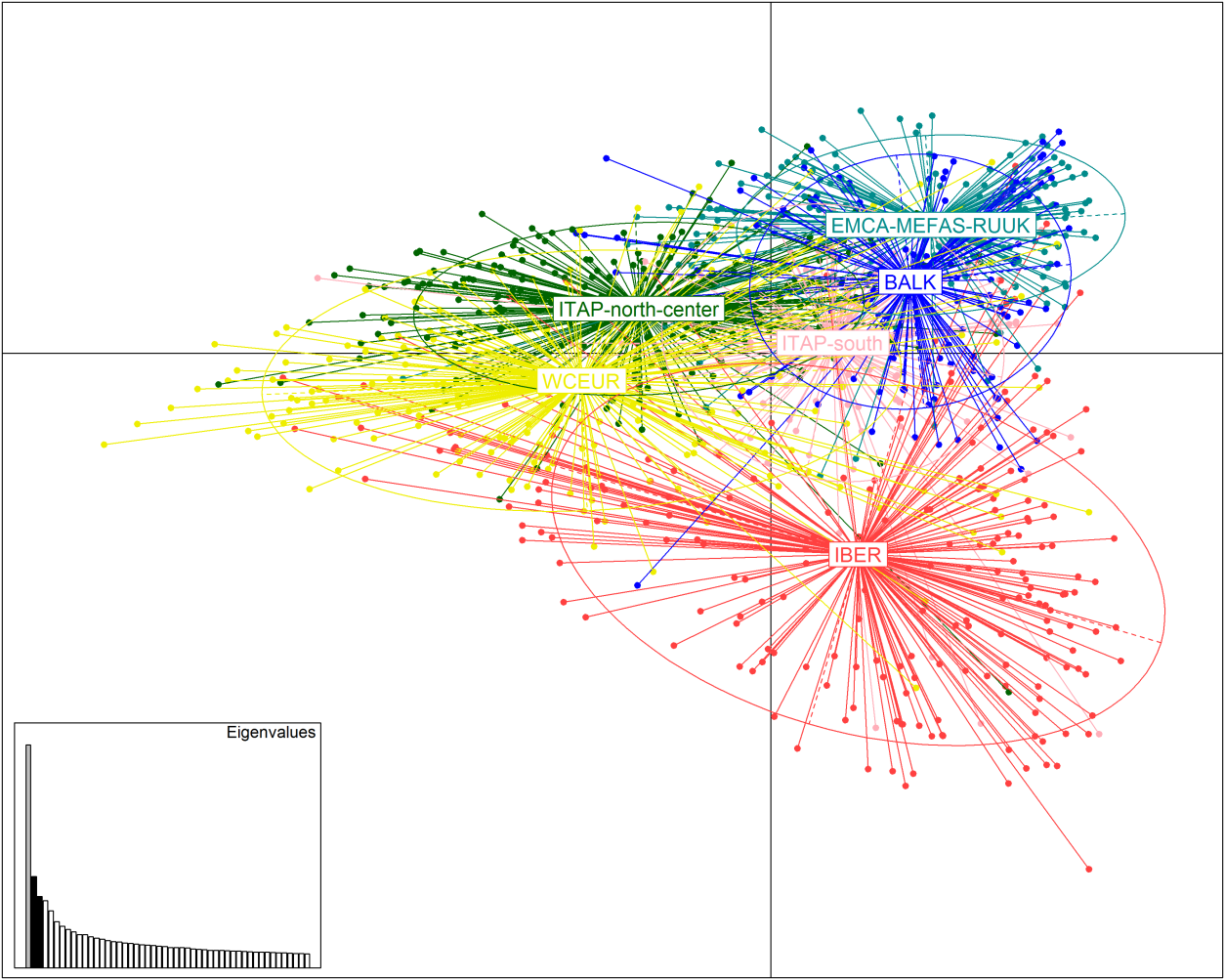
**

**Supplementary Figure S4** Principal Coordinates Analysis (PCoA) on the 1,038 grapevine varieties collected across Europe, Caucasus, Middle and Far East and analyzed by 18k SNP genotyping array. The varieties were highlighted based on their passport (Supplementary Table S1).


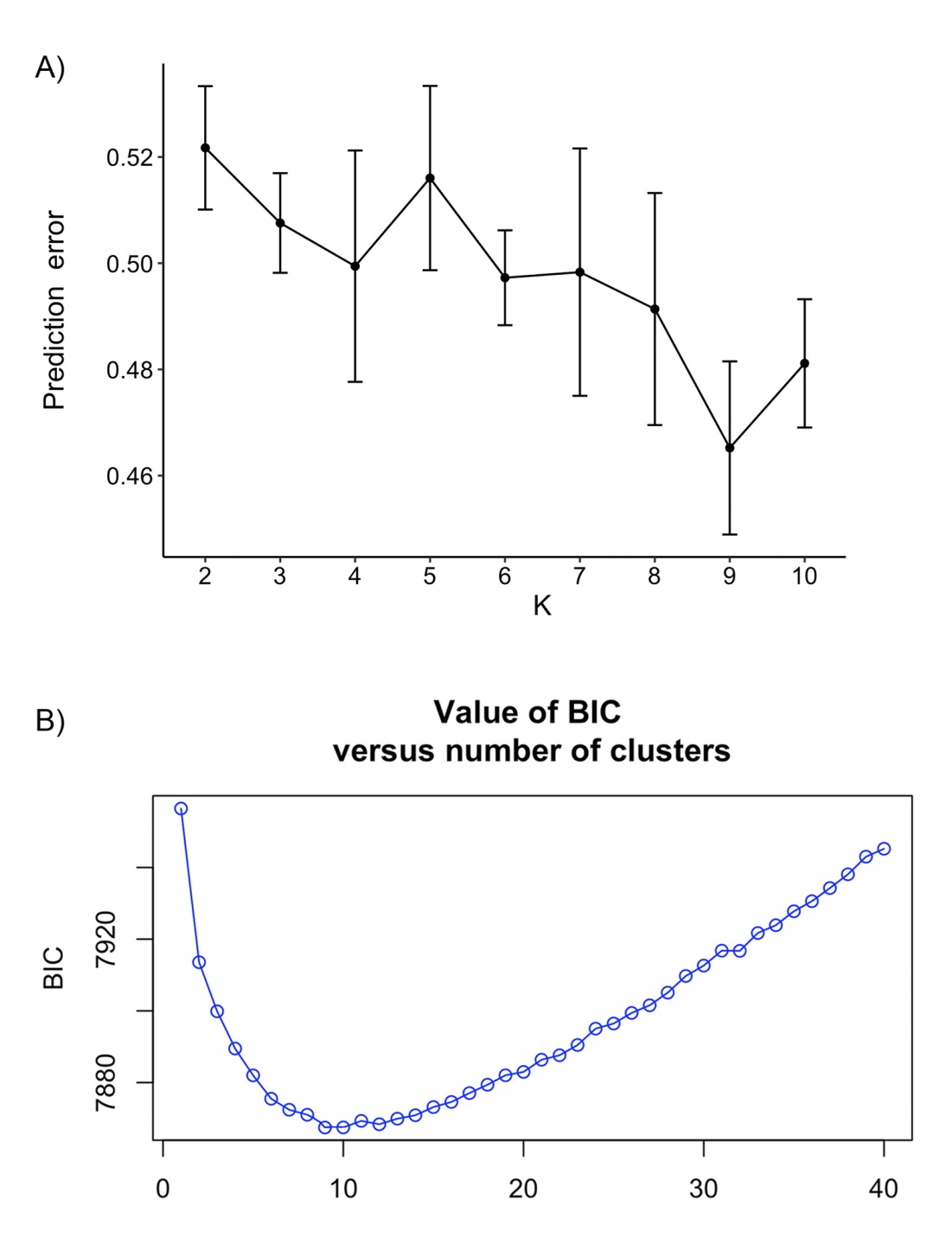


**Supplementary Figure S5** Prediction error (**A**) and Bayesian Information Criterion (BIC) (**B**) evaluated through fastStructure and Discriminant Analysis of Principal Components (DAPC), respectively. The two approaches were used to infer the *K*-means clustering (*K* = 9) within the whole grapevine germplasm (1,038 accessions).


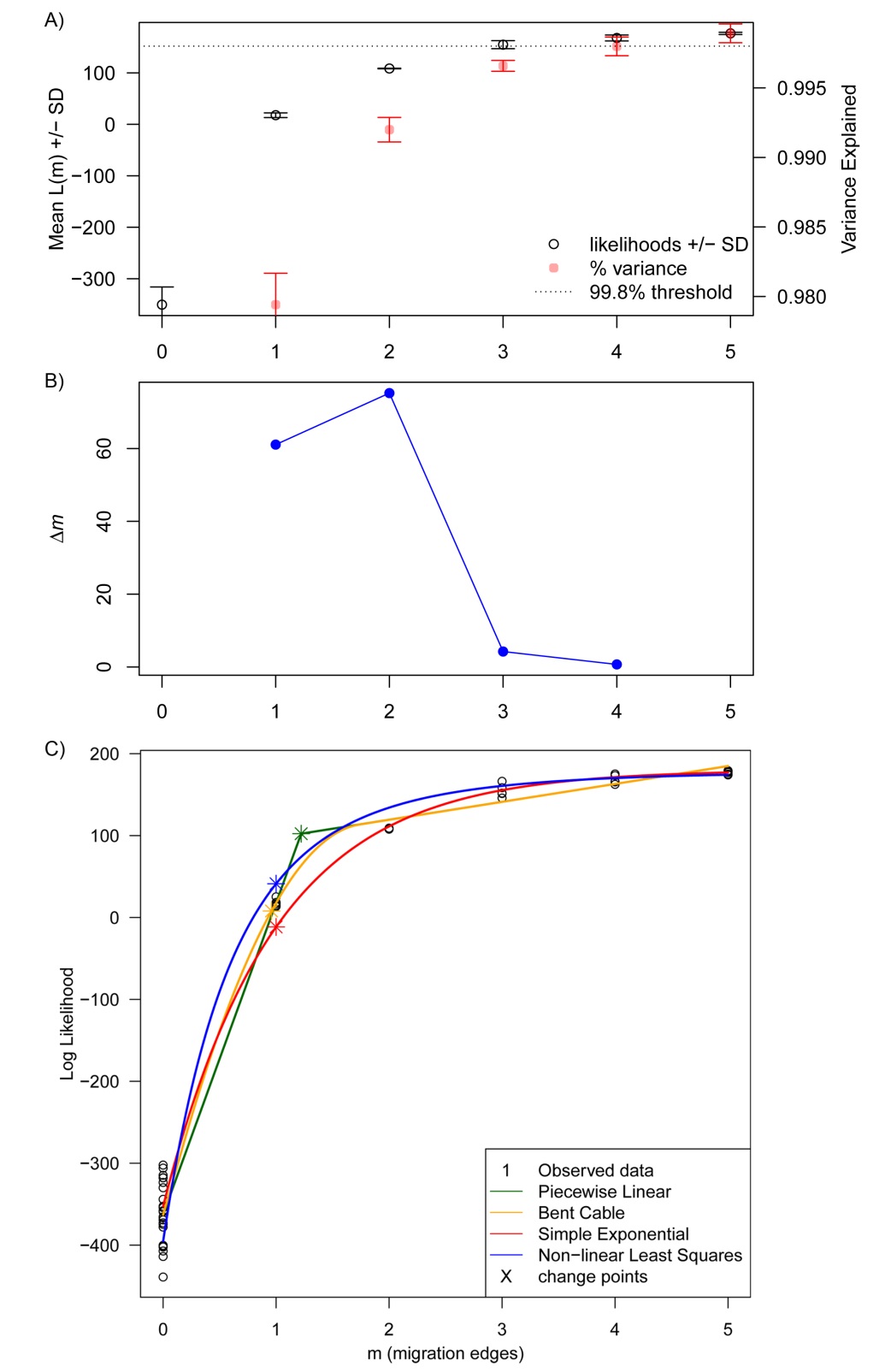


**Supplementary Figure S6** Estimation of the optimal number of migration edges. **A)** Change of likelihood weighted and the variance explained for each *m* evaluated. The best *m* (4) explains 99.8% of variance; **B)** *Δm* evaluated by *ad hoc* statistic based on the Evanno’s method. **C)** Estimation of the optimal *m* based on a piecewise linear (change point), bent cable (alpha), simple exponential (threshold, default 5%), and non-linear least squares (threshold, default 5%) models; both methods underlined the best *m* at 2.


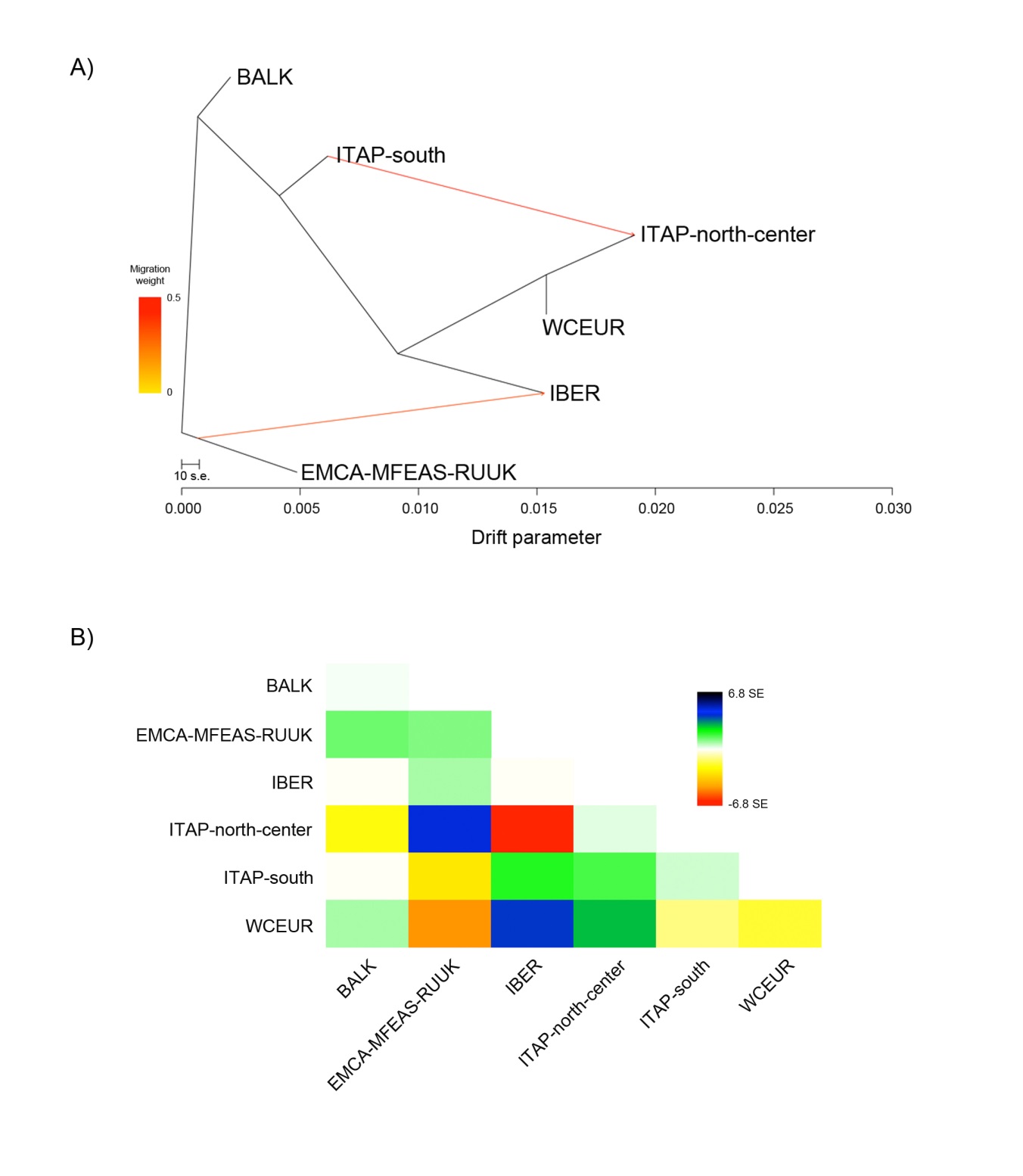


**Supplementary Figure S7** **A)** ML-tree inferring two admixture events (*m* = 2), from ITAP-south to ITAP-north-center (*p* << 0.001) and from EMCA-MFEAS-RUUK to IBER (*p* << 0.001) populations; **B)** scaled residual fit of predicted model, with SE ± 6.8.


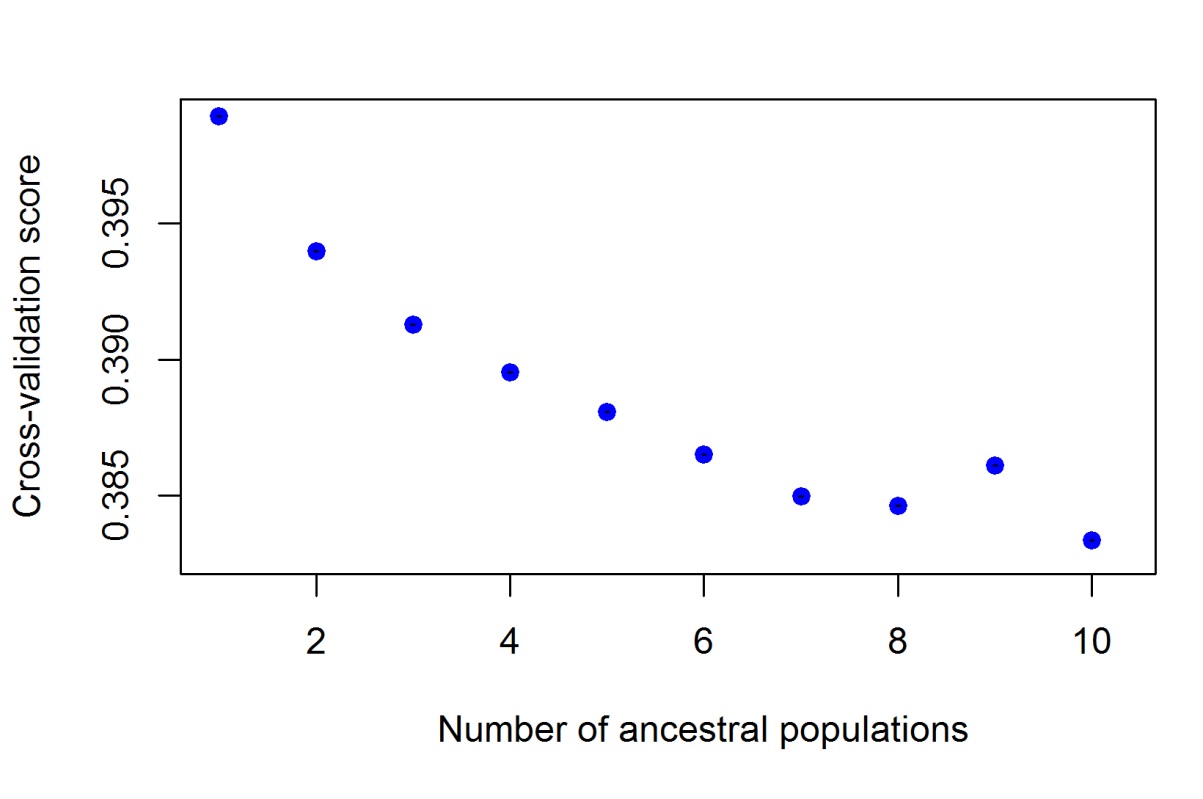


**Supplementary Figure S8** Cross validation score for the grapevine germplasm (1,059 accessions) analyzed by R/TESS3. Ancestry coefficients estimation was performed using *tess3* function, running the program for *K* ranging from 1 to 10. The cross‐entropy criterion compared the genotypic frequencies predicted from the training set to those computed from the test set at each locus. Smaller values of the criterion indicate better estimates for R/TESS3. Therefore, the best choice for *K* corresponds to a plateau or starts increasing in the cross‐entropy plot. After cross‐validation method the best *K=8*.


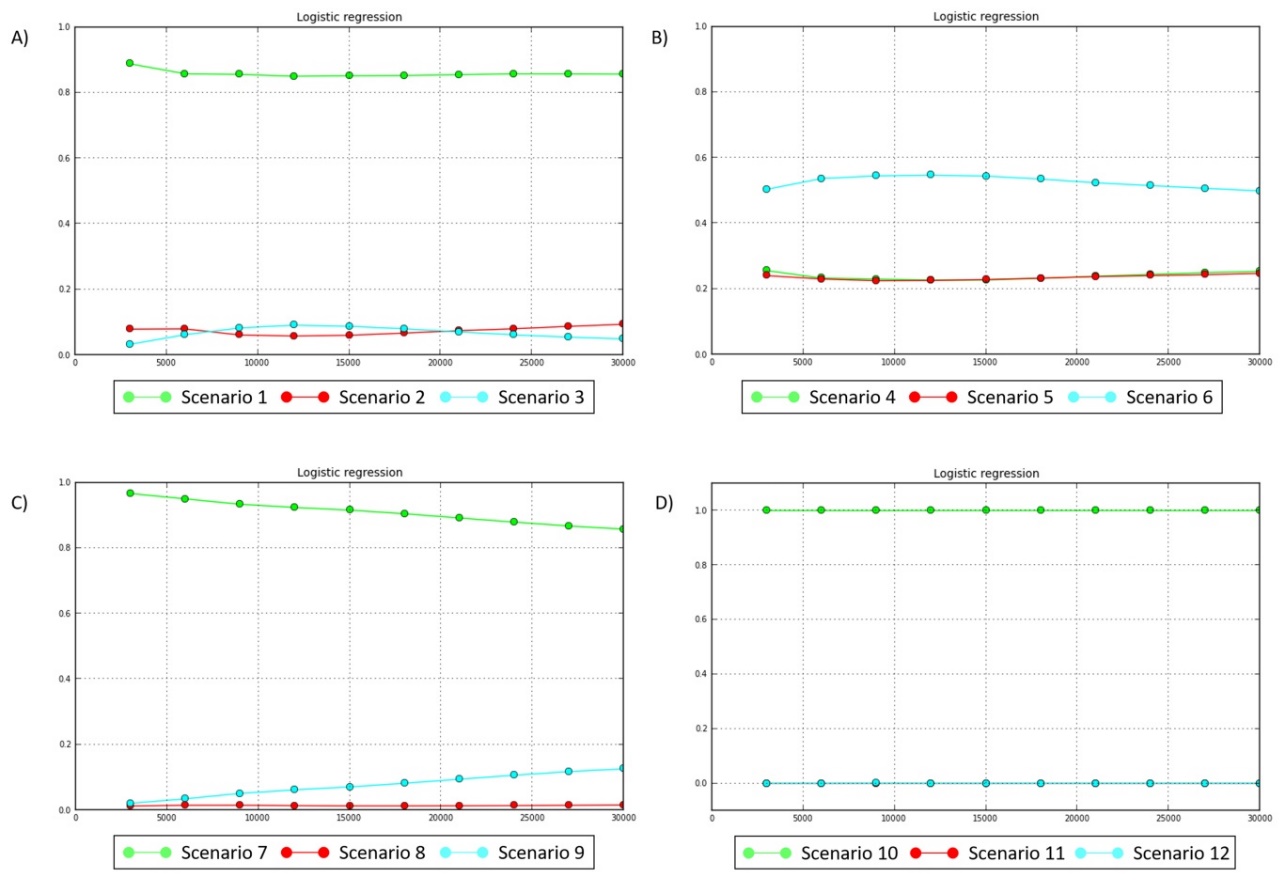


**Supplementary Figure S9** Result of ABC analysis implemented in DIYABC to identify the gene flow of grapevine genetic resources in the Mediterranean Basin. Plotting of logistic regression of each scenario probability. Four groups of hypotheses were evaluated (totaling 12 demographic scenarios): 1) about the origin of Southern Italian genotypes; 2) the gene flow from Italy to Western and Central Europe; 3) the gene flow from Southern Italy to Western and Central Europe through Iberian Peninsula; 4) the gene flow from Middle and Far East to Iberian Peninsula through Northern Africa. **A)** Scenario 1-3, **B)** Scenario 4-6, **C)** Scenario 7-9 and **D)** Scenario 10-12. Scenarios are defined in Fig. 6.


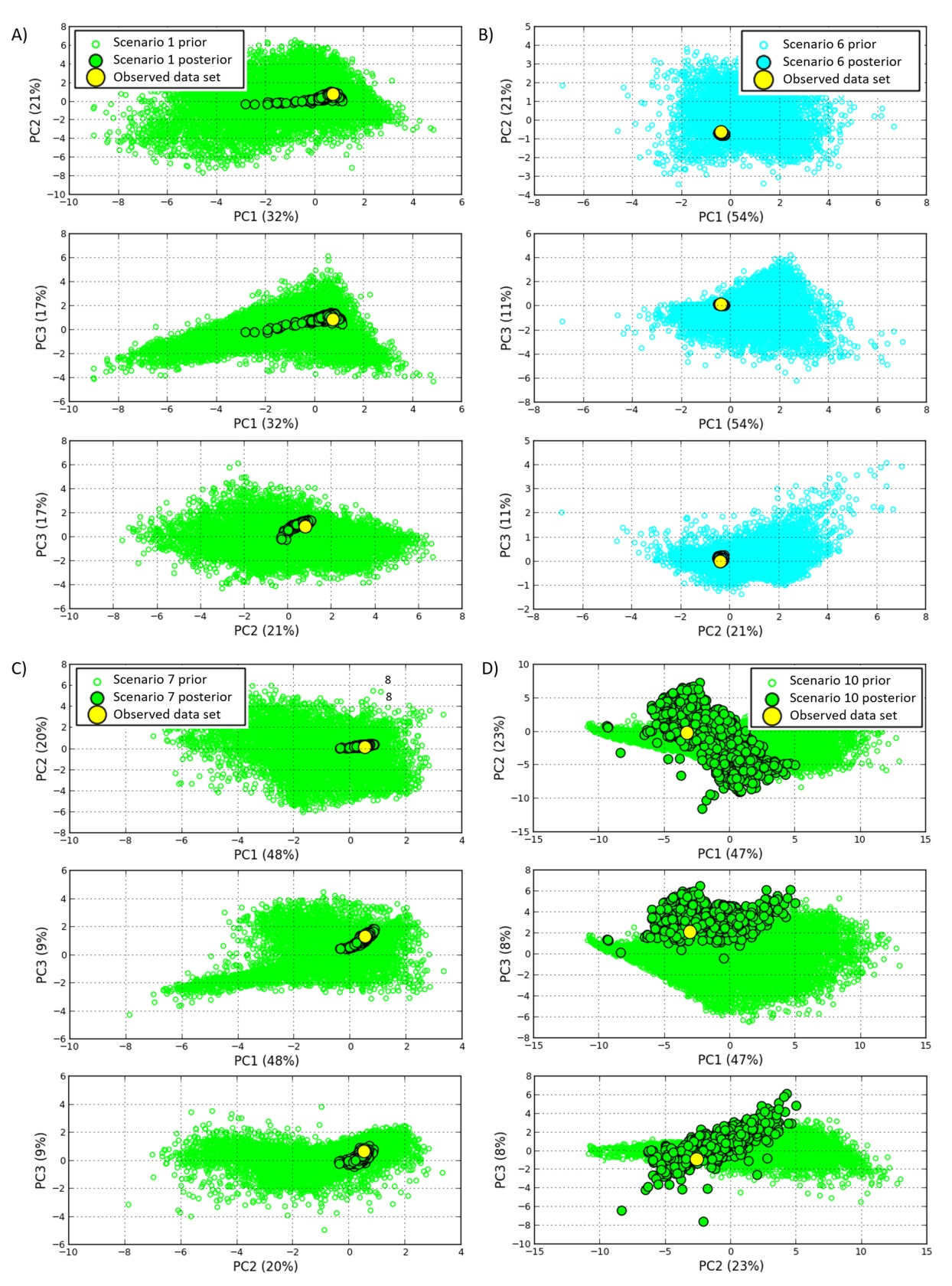


**Supplementary Figure S10** PCA plots using the first three principal components showing datasets simulated from the prior distribution of the parameters (open circles), from the posterior predictive distribution (filled circles), as well as the observed dataset (yellow circle), per each of the four most likely scenarios. **A)** Scenario 1; **B)** Scenario 6; **C)** Scenario 7; **D)** Scenario 10. Each scenario is defined in Fig. 6.
